# Synthesis, In Vitro α-Glucosidase Inhibitory Activity and Molecular Docking Studies of Novel Benzothiazole-Triazole Derivatives

**DOI:** 10.3390/molecules22091555

**Published:** 2017-09-15

**Authors:** Zipeng Gong, Yaping Peng, Jie Qiu, Anbai Cao, Guangcheng Wang, Zhiyun Peng

**Affiliations:** 1 Provincial Key Laboratory of Pharmaceutics in Guizhou Province, Guizhou Medical University, Beijing Road, Guiyang 550004, China; gzp4012607@126.com; 2 School of Pharmacy, Guizhou Medical University, 4 Beijing Road, Guiyang 550004, China; 3 National Engineering Research Center of Miao’s Medicines, 4 Beijing Road, Guiyang 550004, China; 4 College of Chemistry and Chemical Engineering, Hunan Engineering Laboratory for Analyse and Drugs Development of Ethnomedicine in Wuling Mountains, Jishou University, Jishou 416000, China; yapingpeng17@163.com (Y.P.); qiujie_jsu@126.com (J.Q.); AnbaiCao1997@163.com (A.C.)

**Keywords:** benzothiazole, triazole, α-glucosidase, molecular docking

## Abstract

Benzothiazole-triazole derivatives **6a**–**6s** have been synthesized and characterized by ^1^H-NMR and ^13^C-NMR. All synthetic compounds were screened for their in vitro α-glucosidase inhibitory activity by using Baker’s yeast α-glucosidase enzyme. The majority of compounds exhibited a varying degree of α-glucosidase inhibitory activity with IC_50_ values between 20.7 and 61.1 μM when compared with standard acarbose (IC_50_ = 817.38 μM). Among the series, compound **6s** (IC_50_ = 20.7 μM) bearing a chlorine group at the 5-position of the benzothiazole ring and a tert-butyl group at the para position of the phenyl ring, was found to be the most active compound. Preliminary structure-activity relationships were established. Molecular docking studies were performed to predict the binding interaction of the compounds in the binding pocket of the enzyme.

## 1. Introduction

Type 2 diabetes is the most common type of diabetes, accounting for about 95 percent of diabetes cases. It is characterized by high blood glucose (hyperglycemia), insulin resistance and relative lack of insulin [1]. Hyperglycemia plays an important role in the development of type 2 diabetes and complications associated with diseases such as microvascular and macrovascular diseases [2]. α-Glucosidase catalyzes the hydrolysis of terminal 1,4-linked α-d-glucose residues and releases free α-d-glucose, which is to the main cause of hyperglycemia [3]. Inhibition of α-glucosidase activity is one of the important therapeutic approaches for the treatment of type 2 diabetes by slowing the absorption process of glucose in intestine [4]. Three inhibitors of α-glucosidase enzyme (acarbose, miglitol, and voglibose) are being clinically used for the management of type 2 diabetes, and these are also used as anticancer [5], anti-HIV [6], and anti-hepatitis agents [7].

**Figure 1:**
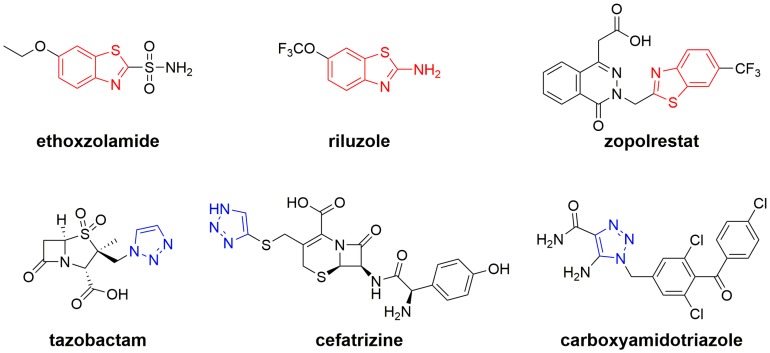
The structures of some commercial drugs containing benzothiazole or 1,2,3-triazole moiety.

Benzothiazole is an important scaffold in bioorganic and medicinal chemistry, and has broad applications in drug discovery and development [8]. Benzothiazole-based compounds exhibit numerous biological activities such as anticonvulsant [9], anti-inflammatory [10], antimicrobial [11], anticancer [12] and anti-diabetic activities [13]. Some of benzothiazole-containing drugs, such as riluzole (anticonvulsant), ethoxzolamide (glaucoma, ulcers and as a diuretic), and zopolrestat (diabetic nephropathy and diabetes), have been used clinically for many years (Figure 1). Recently, M. Taha et al. reported the synthesis of a new class of benzothiazole hybrid with benzohydrazide moiety, and some of these showed superior activity compared to standard acarbose [14].

On the other hand, 1,2,3-triazoles are an important class of heterocyclic compounds, and have been widely applied in various fields, including synthetic organic chemistry, biological science, material science and medicinal chemistry [15,16]. Notably, 1,2,3-triazole derivatives have been reported to exhibit various biological activities such as antioxidant [17], antibacterial [18], antitubercular [19] and anticancer [20]. There are some clinical and commercial drugs, such as tazobactam, cefatrizine and carboxyamidotriazole, with 1,2,3-triazole moiety (Figure 1). In particular, several recent studies have shown that some 1,2,3-triazole derivatives exhibit α-glucosidase inhibitory activity [21,22,23,24,25].

In continuation of our drug discovery research on potential antidiabetic agents [25,26,27,28], we synthesized a novel series of benzothiazole-triazole derivatives and evaluated for their in vitro α-glucosidase inhibitory activity. Furthermore, molecular docking was also performed to predict the binding interaction of the compounds in the binding pocket of the enzyme.

## 2. Results and Discussion

### 2.1. Chemistry

**Scheme 1:**
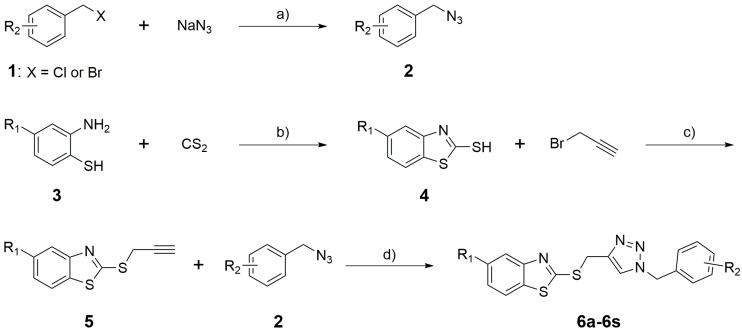
(**a**) DMF, room temperature, 24 h; (**b**) KOH, EtOH/H_2_O, reflux, 12 h; (**c**) K_2_CO_3_, acetone, reflux, 5 h; (**d**) sodium ascorbate, CuSO_4_·5H_2_O, DMF, r.t., 4 h.

The synthesis of benzothiazole-triazole derivatives **6a**–**6s** is shown in Scheme 1. The reaction of various benzyl chlorides or benzyl bromides **1** with NaN_3_ in DMF at room temperature for 24 h provided the corresponding (azidomethyl)aryl **2**. Treatment of substituted 2-aminobenzenethiol with CS_2_ in the presence of KOH in reflux EtOH/H_2_O afforded substituted benzo[*d*]thiazole-2-thiol **4**, which reacted with 3-bromoprop-1-yne to provide the key intermediate **5**. In the final step, according to Huisgen, the 1,3-dipolar cycloaddition reaction (click reaction), condensation of intermediate **5** with various (azidomethyl)aryl **2** in the presence of sodium ascorbate and CuSO_4_·5H_2_O in DMF solution resulted in the formation of the title products **6a**–**6s**.

The structures of all the title compounds **6a**–**6s** were characterized by ^1^H-NMR and ^13^C-NMR spectra (Supplementary Materials). For instance, the ^1^H-NMR spectrum of **6a** shows two singlet signals at δ 4.68 ppm and δ 5.55 ppm, which were assigned to the methylene protons of SCH_2_ and NCH_2_, respectively. A doublet of two protons appeared at δ 7.19 ppm (*J* = 8.4 Hz), which was assigned to the aromatic protons of C2,6-H of the right phenyl ring. The benzothiazole protons appeared as two doublets of one proton each at δ 7.86 and δ 7.99 ppm, and a triplet of one proton at δ 7.37 ppm with a coupling constant of 8.0 Hz and 8.4 Hz, respectively. The rest of the protons of benzothiazole and the right phenyl appeared as a multiplet of three protons between δ 7.45 and 7.51 ppm. The single peak of the C-H of triazole ring was observed at δ 8.17 ppm. The ^13^C-NMR spectrum of **6a** showed two characteristic peaks for methylene carbon at δ 27.9 ppm and δ 52.5 ppm. The remaining thirteen signals were assigned to benzothiazole, triazole and phenyl carbon in the compound **6a**. Therefore, the data of ^1^H-NMR and ^13^C-NMR is in agreement with the structure of compound **6a**.

### 2.2. α-Glucosidase Inhibition Assay

All the synthetic benzothiazole-triazole derivatives (**6a**–**6s**) were screened to evaluate their in vitro α-glucosidase inhibitory activity by using Baker’s yeast α-glucosidase enzyme. The results are summarized in Table 1. The majority of compounds exhibited a varying degree of α-glucosidase inhibitory activity with IC_50_ values between 20.7 and 61.1 μM when compared with standard acarbose (IC_50_ = 817.38 μM). Among the series, compounds **6a**, **6e**, **6i**, **6m**, **6n**, **6o** and **6s** displayed potent inhibitory activity with IC_50_ values of 28.7, 27.4, 29.4, 28.2, 28.0, 22.3 and 20.7 μM.

### 2.3. Structure-Activity Relationship

The structure-activity relationship of this class of compounds has been summarized. The introduction of electron withdrawing groups such as fluorine, chlorine, and bromine into the phenyl ring results in an increase in inhibitory activity. Furthermore, shifting these groups from the para to the ortho position decreased inhibitory activity. Introduction of the electron-withdrawing group chlorine into the benzothiazole ring results in a significant increase in inhibitory activity. It is interesting to point out that **6i** and **6s** containing the tert-butyl group at the para position of the phenyl ring exhibited potent α-glucosidase inhibitory activity, with IC_50_ values of 29.4 and 20.7 μM, respectively. In particular, compound **6s** with a chlorine group at the 5-position of the benzothiazole ring and a tert-butyl group at the para position of the phenyl ring, was found to be the most active compound in this series. In order to illustrate the binding interactions of molecules into the active site of α-glucosidase, molecular docking studies were conducted.

### 2.4. Molecular Docking

**Figure 2:**
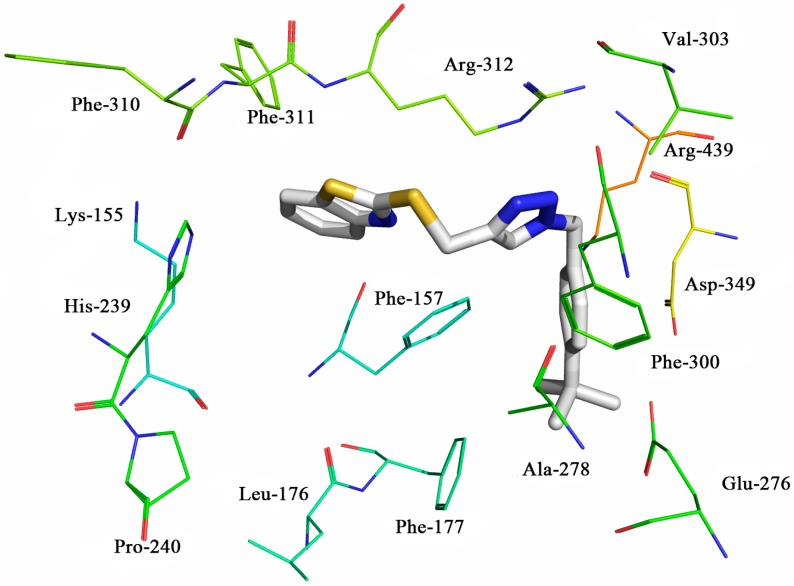
Compound **6i** was docked to the binding pocket of the *Saccharomyces cerevisiae* α-glucosidase.

The theoretical binding mode between **6i** and *Saccharomyces cerevisiae* α-glucosidase is shown in Figure 2. Compound **6i** adopted an “L-shaped” conformation in the pocket of the α-glucosidase. The benzothiazole group of **6i** was located at the hydrophobic pocket, surrounded by the residues Phe-157, Phe-310 and Phe-311, while the 4-(tert-butyl)phenyl group of **6i** stretched into the hydrophobic pocket consisting of Phe-157, Phe-177 and Phe-300, forming a stable hydrophobic binding. Detailed analysis showed that the triazole group in the middle of **6i** formed CH-π interaction with the residue Phe-300. In addition, cation-π interactions were observed between **6i** and the residues Lys-155, Arg-312 and Arg-439. Furthermore, **6i** formed anion-π interactions with the residues Glu-276 and Asp-349, respectively. All these interactions helped **6i** to anchor in the binding site of the α-glucosidase.

**Figure 3:**
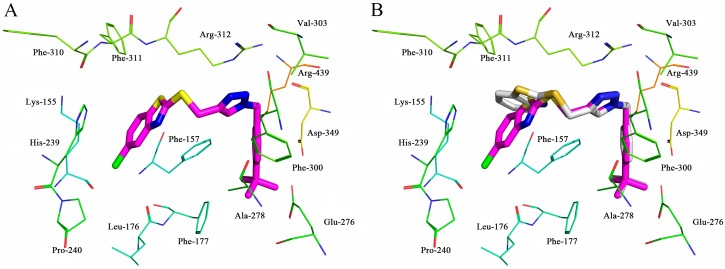
(**A**) Compound **6s** was docked to the binding pocket of the *Saccharomyces cerevisiae* α-glucosidase; (**B**) Compounds **6i** and **6s** were docked to the binding pocket of the *Saccharomyces cerevisiae* α-glucosidase (overlapped).

To explain the activity order of **6i** and **6s** against α-glucosidase, **6s** was then docked to the binding site of α-glucosidase; the theoretical binding mode between **6s** and α-glucosidase is shown in Figure 3A. The interaction between **6s** and α-glucosidase was almost the same as the precursor **6i**. The only difference was that the benzothiazole group of **6s** formed π-π stacking interactions with the residues His-239 and Phe-157, which were not formed by **6i**, making **6s** more active than **6i** against α-glucosidase (Figure 3B). In addition, the estimated binding energies were −8.6 kcal·mol^−1^ for **6i** and −8.9 kcal·mol^−1^ for **6s**, respectively, which was consistent with the results of the in vitro anti-α-glucosidase assay. In summary, the above molecular simulations give us rational explanation of the interactions between **6i**, **6s** and α-glucosidase, which provided valuable information for further development of α-glucosidase inhibitors.

## 3. Experimental Section

### 3.1. General

All starting materials and reagents were purchased from commercial suppliers. Nuclear magnetic resonance spectra (NMR) were recorded on a Bruker spectrometer (400 MHz) with TMS as an external reference and reported in parts per million.

### 3.2. General Procedure for the Synthesis of ***2***

A mixture of substituted benzyl chlorides or benzyl bromides **1** (1.0 mmol) and NaN_3_ (1.2 mmol) in 10 mL DMF was stirred at room temperature for 24 h. After the completion of the reaction (monitored by TLC), the mixture was poured into water and extracted 3 times for ethyl acetate. The combined organic layers were dried over Na_2_SO_4_ and concentrated under vacuum. The residue was purified by chromatography to give **2**.

### 3.3. General Procedure for the Synthesis of ***4***

To a solution of substituted 2-aminobenzenethiol (2 mmol) in EtOH (20 mL) was added CS_2_ (4 mmol) and KOH (4 mmol), and the mixture was stirred at 80 °C for 12 h. After cooling to the room temperature, the mixture was treated with ice-water (100 mL) and 10% HCl was added to adjust pH to 2–3. The solid precipitate was collected by filtration to obtain **4**, which was used in the next step without further purification.

### 3.4. General Procedure for the Synthesis of ***5***

A mixture of **4** (1 mmol), 3-bromoprop-1-yne (1.5 mmol) and K_2_CO_3_ (3 mmol) in acetone (20 mL) was stirred at reflux for 5 h. The solvent was evaporated under reduced pressure and the residue was purified by chromatography to give **5**.

### 3.5. General Procedure for the Synthesis of Benzothiazole-Triazole Derivatives (***6a***–***6s***)

A mixture of **5** (1.0 mmol), **2** (1.0 mmol), CuSO_4_·5H_2_O (0.025 g; 0.1 mmol) and sodium ascorbate (0.10 g, 0.5 mmol) in DMF (10 mL) was stirred at room temperature for 4 h. After completion of reaction, the content was poured into 50 mL ice cold water and extracted with ethylacetate (50 mL × 3). The combined organic extracts were dried over Na_2_SO_4_, filtered, and concentrated. The residue was purified by chromatography on silica gel with EtOAc/petroleum ether to give the title products **6a**–**6s**.

*2-(((1-(4-Bromobenzyl)-1H-1,2,3-triazol-4-yl)methyl)thio)benzo[d]thiazole* (**6a**). Yellow solid, yield 65%, m.p. 112–114 °C. ^1^H-NMR (*d*_6_-DMSO, 400 MHz) δ: 4.68 (s, 2H, SCH_2_), 5.55 (s, 2H, NCH_2_), 7.19 (d, 2H, *J* = 8.4 Hz, ArH), 7.37 (t, 1H, *J* = 8.4 Hz, ArH), 7.45–7.51 (m, 3H, ArH), 7.86 (d, 1H, *J* = 8.0 Hz, ArH), 7.99 (d, 1H, *J* = 8.0 Hz, ArH), 8.17 (s, 1H, CH-triazole); ^13^C-NMR (*d*_6_-DMSO, 100 MHz) δ: 27.9, 52.5, 121.7, 121.8, 122.3, 124.6, 125.0, 126.8, 130.5, 132.1, 135.2, 135.8, 143.2, 153.0, 166.1; MS (ESI, *m*/*z*): 416.96 [M + H]^+^.

*2-(((1-(2-Chlorobenzyl)-1H-1,2,3-triazol-4-yl)methyl)thio)benzo[d]thiazole* (**6b**). Yellow solid, yield 79%, m.p. 110–111 °C. ^1^H-NMR (*d*_6_-DMSO, 400 MHz) δ: 4.69 (s, 2H, SCH_2_), 5.66 (s, 2H, NCH_2_), 7.11 (dd, 1H, *J* = 8.0 Hz, 1.2 Hz, ArH), 7.27 (dt, 1H, *J* = 8.0 Hz, 1.2 Hz, ArH), 7.33–7.39 (m, 2H, ArH), 7.45–7.49 (m, 2H, ArH), 7.86 (d, 1H, *J* = 8.0 Hz, ArH), 7.99 (d, 1H, *J* = 8.0 Hz, ArH), 8.15 (s, 1H, CH-triazole); ^13^C-NMR (*d*_6_-DMSO, 100 MHz) δ: 27.9, 51.1, 121.7, 122.3, 125.0, 125.0, 126.8, 128.1, 130.0, 130.6, 130.7, 133.0, 133.7, 135.2, 143.0, 153.0, 166.1; MS (ESI, *m*/*z*): 373.03 [M + H]^+^.

*2-(((1-(2-Bromobenzyl)-1H-1,2,3-triazol-4-yl)methyl)thio)benzo[d]thiazole* (**6c**). Yellow solid, yield 52%, m.p. 104–106 °C. ^1^H-NMR (*d*_6_-DMSO, 400 MHz) δ: 4.70 (s, 2H, SCH_2_), 5.64 (s, 2H, NCH_2_), 7.05 (dd, 1H, *J* = 8.0 Hz, 1.2 Hz, ArH), 7.24–7.33 (m, 2H, ArH), 7.37 (dt, 1H, *J* = 8.0 Hz, 1.2 Hz, ArH), 7.47 (dt, 1H, *J* = 8.0 Hz, 1.2 Hz, ArH), 7.62 (dd, 1H, *J* = 8.0 Hz, 1.2 Hz, ArH), 7.86 (d, 1H, *J* = 8.0 Hz, ArH), 7.99 (d, 1H, *J* = 8.0 Hz, ArH), 8.14 (s, 1H, CH-triazole); ^13^C-NMR (*d*_6_-DMSO, 100 MHz) δ: 27.9, 53.4, 121.7, 122.3, 123.2, 125.0, 125.0, 126.8, 128.6, 130.7, 130.8, 133.3, 135.2, 135.3, 143.0, 153.0, 166.0; MS (ESI, *m*/*z*): 416.98 [M + H]^+^.

*2-(((1-(3-Fluorobenzyl)-1H-1,2,3-triazol-4-yl)methyl)thio)benzo[d]thiazole* (**6d**). White solid, yield 67%, m.p. 112–114 °C. ^1^H-NMR (*d*_6_-DMSO, 400 MHz) δ: 4.69 (s, 2H, SCH_2_), 5.59 (s, 2H, NCH_2_), 7.06–7.16 (m, 3H, ArH), 7.33–7.38 (m, 2H, ArH), 7.47 (dt, 1H, *J* = 8.0 Hz, 1.2 Hz, ArH), 7.87 (d, 1H, *J* = 8.0 Hz, ArH), 7.99 (d, 1H, *J* = 8.0 Hz, ArH), 8.22 (s, 1H, CH-triazole); ^13^C-NMR (*d*_6_-DMSO, 100 MHz) δ: 27.9, 52.6, 115.1 (d, 1C, *J* = 21.0 Hz), 115.3 (d, 1C, *J* = 21.0 Hz), 121.7, 122.3, 124.3 (d, 1C, *J* = 2.8 Hz), 124.7, 125.0, 126.8, 131.2 (d, 1C, *J* = 8.3 Hz), 135.2, 139.1 (d, 1C, *J* = 7.5 Hz), 143.2, 153.0, 161.3 (d, 1C, *J* = 243 Hz), 166.1; MS (ESI, *m*/*z*): 357.06 [M + H]^+^.

*2-(((1-(4-Chlorobenzyl)-1H-1,2,3-triazol-4-yl)methyl)thio)benzo[d]thiazole* (**6e**). White solid, yield 81%, m.p. 119–120 °C. ^1^H-NMR (*d*_6_-DMSO, 400 MHz) δ: 4.68 (s, 2H, SCH_2_), 5.57 (s, 2H, NCH_2_), 7.25 (d, 2H, *J* = 8.4 Hz, ArH), 7.36 (d, 2H, *J* = 8.4 Hz, ArH), 7.40 (dd, 1H, *J* = 8.4 Hz, 2.0 Hz, ArH), 7.96 (d, 1H, *J* = 2.0 Hz, ArH), 8.03 (d, 1H, *J* = 8.4 Hz, ArH), 8.20 (s, 1H, CH-triazole); ^13^C-NMR (*d*_6_-DMSO, 100 MHz) δ: 28.0, 52.5, 121.2, 123.7, 124.8, 125.0, 129.1, 130.2, 131.7, 133.3 134.0, 135.4, 143.0, 153.9, 169.0; MS (ESI, *m*/*z*): 373.03 [M + H]^+^.

*2-(((1-(2-Fluorobenzyl)-1H-1,2,3-triazol-4-yl)methyl)thio)benzo[d]thiazole* (**6f**). Yellow solid, yield 64%, m.p. 86–88 °C. ^1^H-NMR (*d*_6_-DMSO, 400 MHz) δ: 4.69 (s, 2H, SCH_2_), 5.63 (s, 2H, NCH_2_), 7.12–7.28 (m, 3H, ArH), 7.35–7.39 (m, 2H, ArH), 7.47 (dt, 1H, *J* = 8.0 Hz, 1.2 Hz, ArH), 7.86 (d, 1H, *J* = 8.0 Hz, ArH), 7.99 (d, 1H, *J* = 8.0 Hz, ArH), 8.16 (s, 1H, CH-triazole); ^13^C-NMR (*d*_6_-DMSO, 100 MHz) δ: 27.9, 47.3 (d, 1C, *J* = 3.8 Hz), 115.9 (d, 1C, *J* = 20.8 Hz), 121.7, 122.3, 123.2 (d, 1C, *J* = 14.6 Hz), 124.7, 125.0, 125.2 (d, 1C, *J* = 3.5 Hz), 126.8, 131.0 (d, 1C, *J* = 3.5 Hz), 131.1 (d, 1C, *J* = 8.2 Hz), 135.2, 143.1, 153.0, 159.3 (d, 1C, *J* = 245 Hz), 166.1; MS (ESI, *m*/*z*): 357.06 [M + H]^+^.

*2-(((1-Benzyl-1H-1,2,3-triazol-4-yl)methyl)thio)benzo[d]thiazole* (**6g**) [29]. White solid, yield 72%, m.p. 109–110 °C. ^1^H-NMR (*d*_6_-DMSO, 400 MHz) δ: 4.68 (s, 2H, SCH_2_), 5.56 (s, 2H, NCH_2_), 7.23–7.25 (m, 2H, ArH), 7.28–7.31 (m, 3H, ArH), 7.37 (dt, 1H, *J* = 8.0 Hz, 1.2 Hz, ArH), 7.47 (dt, 1H, *J* = 8.0 Hz, 1.2 Hz, ArH), 7.87 (d, 1H, *J* = 8.0 Hz, ArH), 8.00 (d, 1H, *J* = 8.0 Hz, ArH), 8.18 (s, 1H, CH-triazole); ^13^C-NMR (*d*_6_-DMSO, 100 MHz) δ: 27.9, 53.2, 121.7, 122.3, 124.6, 125.0, 126.8, 128.3, 128.5, 129.2, 135.2, 136.4, 143.1, 153.1, 166.1; MS (ESI, *m*/*z*): 339.07 [M + H]^+^.

*2-(((1-(3,5-Dichlorobenzyl)-1H-1,2,3-triazol-4-yl)methyl)thio)benzo[d]thiazole* (**6h**). White solid, yield 75%, m.p. 85–87 °C. ^1^H-NMR (*d*_6_-DMSO, 400 MHz) δ: 4.69 (s, 2H, SCH_2_), 5.65 (s, 2H, NCH_2_), 7.15 (d, 1H, *J* = 8.0 Hz, ArH), 7.35–7.39 (m, 2H, ArH), 7.47 (dt, 1H, *J* = 8.0 Hz, 1.2 Hz, ArH), 7.64 (d, 1H, *J* = 2.0 Hz, ArH), 7.86 (d, 1H, *J* = 8.0 Hz, ArH), 8.00 (d, 1H, *J* = 8.0 Hz, ArH), 8.15 (s, 1H, CH-triazole); ^13^C-NMR (*d*_6_-DMSO, 100 MHz) δ: 27.9, 50.5, 121.7, 122.3, 125.0, 126.8, 128.3, 129.6, 132.2, 132.8, 134.1, 134.4, 135.2, 143.1, 153.0, 166.0; MS (ESI, *m*/*z*): 406.99 [M + H]^+^.

*2-(((1-(4-(Tert-butyl)benzyl)-1H-1,2,3-triazol-4-yl)methyl)thio)benzo[d]thiazole* (**6i**). Yellow solid, yield 51%, m.p. 77–78 °C. ^1^H-NMR (*d*_6_-DMSO, 400 MHz) δ: 1.22 (s, 9H, CH_3_), 4.68 (s, 2H, SCH_2_), 5.51 (s, 2H, NCH_2_), 7.15 (d, 2H, *J* = 8.4 Hz, ArH), 7.29 (d, 2H, *J* = 8.4 Hz, ArH), 7.37 (t, 1H, *J* = 8.0 Hz, ArH), 7.47 (t, 1H, *J* = 8.0 Hz, ArH), 7.87 (d, 1H, *J* = 8.0 Hz, ArH), 7.99 (d, 1H, *J* = 8.0 Hz, ArH), 8.16 (s, 1H, CH-triazole); ^13^C-NMR (*d*_6_-DMSO, 100 MHz) δ: 27.9, 31.5, 34.7, 53.0, 121.7, 122.3, 124.5, 125.0, 125.9, 126.8, 128.1, 133.5, 135.2, 143.1, 151.0, 153.1, 166.1; MS (ESI, *m*/*z*): 395.13 [M + H]^+^.

*5-Chloro-2-(((1-(2-fluorobenzyl)-1H-1,2,3-triazol-4-yl)methyl)thio)benzo[d]thiazole* (**6j**). White solid, yield 63%, m.p. 115–117 °C. ^1^H-NMR (*d*_6_-DMSO, 400 MHz) δ: 4.68 (s, 2H, SCH_2_), 5.63 (s, 2H, NCH_2_), 7.12–7.27 (m, 3H, ArH), 7.35–7.40 (m, 1H, ArH), 7.40 (dd, 1H, *J* = 8.0 Hz, 2.0 Hz, ArH), 7.94 (d, 1H, *J* = 2.0 Hz, ArH), 8.03 (d, 1H, *J* = 8.0 Hz, ArH), 8.17 (s, 1H, CH-triazole); ^13^C-NMR (*d*_6_-DMSO, 100 MHz) δ: 28.0, 47.3 (d, 1C, *J* = 3.9 Hz), 115.9 (d, 1C, *J* = 20.8 Hz), 121.2, 123.2 (d, 1C, *J* = 14.6 Hz), 123.7, 124.8, 125.0, 125.2 (d, 1C, *J* = 3.6 Hz), 131.0 (d, 1C, *J* = 3.5 Hz), 131.9 (d, 1C, *J* = 8.2 Hz), 131.6, 134.0, 142.9, 153.9, 159.2 (d, 1C, *J* = 245 Hz), 169.0; MS (ESI, *m*/*z*): 391.02 [M + H]^+^.

*2-(((1-(2-Bromobenzyl)-1H-1,2,3-triazol-4-yl)methyl)thio)-5-chlorobenzo[d]thiazole* (**6k**). White solid, yield 79%, m.p. 119–120 °C. ^1^H-NMR (*d*_6_-DMSO, 400 MHz) δ: 4.69 (s, 2H, SCH_2_), 5.64 (s, 2H, NCH_2_), 7.05 (dd, 1H, *J* = 8.0 Hz, 2.0 Hz, ArH), 7.24–7.33 (m, 2H, ArH), 7.40 (dd, 1H, *J* = 8.0 Hz, 2.0 Hz, ArH), 7.61 (dd, 1H, *J* = 8.0 Hz, 1.2 Hz, ArH), 7.93 (d, 1H, *J* = 2.0 Hz, ArH), 8.02 (d, 1H, *J* = 8.8 Hz, ArH), 8.16 (s, 1H, CH-triazole); ^13^C-NMR (*d*_6_-DMSO, 100 MHz) δ: 28.0, 53.4, 121.2, 123.2, 123.7, 125.0, 125.2, 128.6, 130.7, 130.8, 131.6, 133.3, 134.0, 135.3, 142.8, 153.9, 168.9; MS (ESI, *m*/*z*): 450.93 [M + H]^+^.

*5-Chloro-2-(((1-(2-chlorobenzyl)-1H-1,2,3-triazol-4-yl)methyl)thio)benzo[d]thiazole* (**6l**). White solid, yield 66%, m.p. 128–130 °C. ^1^H-NMR (*d*_6_-DMSO, 400 MHz) δ: 4.69 (s, 2H, SCH_2_), 5.66 (s, 2H, NCH_2_), 7.11 (d, 1H, *J* = 8.0 Hz, ArH), 7.27 (t, 1H, *J* = 8.0 Hz, ArH), 7.36 (dt, 1H, *J* = 8.0 Hz, 2.0 Hz, ArH), 7.40 (dd, 1H, *J* = 8.8 Hz, 2.0 Hz, ArH), 7.45 (d, 1H, *J* = 8.0 Hz, ArH), 7.94 (d, 1H, *J* = 2.0 Hz, ArH), 8.03 (d, 1H, *J* = 8.8 Hz, ArH), 8.16 (s, 1H, CH-triazole); ^13^C-NMR (*d*_6_-DMSO, 100 MHz) δ: 28.0, 51.1, 121.2, 123.7, 125.0, 125.2, 128.1, 130.0, 130.6, 130.7, 131.6, 133.0, 133.7, 134.0, 142.8, 153.9, 168.9; MS (ESI, *m*/*z*): 406.99 [M + H]^+^.

*5-Chloro-2-(((1-(4-chlorobenzyl)-1H-1,2,3-triazol-4-yl)methyl)thio)benzo[d]thiazole* (**6m**). White solid, yield 78%, m.p. 111–112 °C. ^1^H-NMR (*d*_6_-DMSO, 400 MHz) δ: 4.68 (s, 2H, SCH_2_), 5.57 (s, 2H, NCH_2_), 7.25 (d, 2H, *J* = 8.0 Hz, ArH), 7.36 (d, 2H, *J* = 8.0 Hz, ArH), 7.40 (dd, 1H, *J* = 8.8 Hz, 2.0 Hz, ArH), 7.95 (d, 1H, *J* = 2.0 Hz, ArH), 8.02 (d, 1H, *J* = 8.4 Hz, ArH), 8.20 (s, 1H, CH-triazole); ^13^C-NMR (*d*_6_-DMSO, 100 MHz) δ: 28.0, 52.4, 121.2, 123.7, 124.8, 125.0, 129.1, 130.2, 131.6, 133.3, 134.0, 135.4, 143.1, 153.9, 169.0; MS (ESI, *m*/*z*): 406.99 [M + H]^+^.

*2-(((1-(4-Bromobenzyl)-1H-1,2,3-triazol-4-yl)methyl)thio)-5-chlorobenzo[d]thiazole* (**6n**). White solid, yield 73%, m.p. 114–116 °C. ^1^H-NMR (*d*_6_-DMSO, 400 MHz) δ: 4.68 (s, 2H, SCH_2_), 5.55 (s, 2H, NCH_2_), 7.19 (d, 2H, *J* = 8.4 Hz, ArH), 7.41 (dd, 1H, *J* = 8.4 Hz, 2.0 Hz, ArH), 7.49 (d, 2H, *J* = 8.4 Hz, ArH), 7.96 (d, 1H, *J* = 2.0 Hz, ArH), 8.03 (d, 1H, *J* = 8.4 Hz, ArH), 8.21 (s, 1H, CH-triazole); ^13^C-NMR (*d*_6_-DMSO, 100 MHz) δ: 28.0, 52.5, 121.2, 121.8, 123.7, 124.8, 125.0, 130.5, 131.6, 132.1, 134.0, 135.9, 1443.1, 153.9, 169.0; MS (ESI, *m*/*z*): 452.94 [M + H]^+^.

*5-Chloro-2-(((1-(3,5-dichlorobenzyl)-1H-1,2,3-triazol-4-yl)methyl)thio)benzo[d]thiazole* (**6o**). Yellow solid, yield 55%, m.p. 124–126 °C. ^1^H-NMR (*d*_6_-DMSO, 400 MHz) δ: 4.69 (s, 2H, SCH_2_), 5.65 (s, 2H, NCH_2_), 7.15 (d, 1H, *J* = 8.4 Hz, ArH), 7.36 (dd, 1H, *J* = 8.4 Hz, 2.0 Hz, ArH), 7.41 (dd, 1H, *J* = 8.4 Hz, 2.0 Hz, ArH), 7.63 (d, 1H, *J* = 2.0 Hz, ArH), 7.94 (d, 1H, *J* = 2.0 Hz, ArH), 8.03 (d, 1H, *J* = 8.4 Hz, ArH), 8.17 (s, 1H, CH-triazole); ^13^C-NMR (*d*_6_-DMSO, 100 MHz) δ: 27.9, 50.5, 121.2, 123.7, 125.0, 125.2, 128.2, 129.5, 131.6, 132.1, 132.9, 134.0, 134.1, 134.4, 142.9, 153.9, 168.9; MS (ESI, *m*/*z*): 440.95 [M + H]^+^.

*5-Chloro-2-(((1-(3-fluorobenzyl)-1H-1,2,3-triazol-4-yl)methyl)thio)benzo[d]thiazole* (**6p**). White solid, yield 74%, m.p. 140–141 °C. ^1^H-NMR (*d*_6_-DMSO, 400 MHz) δ: 4.68 (s, 2H, SCH_2_), 5.59 (s, 2H, NCH_2_), 7.06–7.14 (m, 3H, ArH), 7.34–7.36 (m, 1H, ArH), 7.40 (dd, 1H, *J* = 8.4 Hz, 2.0 Hz, ArH), 7.95 (d, 1H, *J* = 2.0 Hz, ArH), 8.02 (d, 1H, *J* = 8.4 Hz, ArH), 8.23 (s, 1H, CH-triazole); ^13^C-NMR (*d*_6_-DMSO, 100 MHz) δ: 28.0, 52.6, 115.0 (d, 1C, *J* = 21.9 Hz), 115.3 (d, 1C, *J* = 20.8 Hz), 121.2, 123.7, 124.3 (d, 1C, *J* = 2.8 Hz), 124.9, 125.0, 131.2 (d, 1C, *J* = 5.3 Hz), 131.7, 134.0, 139.1 (d, 1C, *J* = 7.5 Hz), 143.1, 153.9, 161.3 (d, 1C, *J* = 243 Hz), 169.0; MS (ESI, *m*/*z*): 391.02 [M + H]^+^.

*5-Chloro-2-(((1-(4-fluorobenzyl)-1H-1,2,3-triazol-4-yl)methyl)thio)benzo[d]thiazole* (**6q**). Yellow solid, yield 68%, m.p. 108–109 °C. ^1^H-NMR (*d*_6_-DMSO, 400 MHz) δ: 4.68 (s, 2H, SCH_2_), 5.55 (s, 2H, NCH_2_), 7.14 (d, 2H, *J* = 8.8 Hz, ArH), 7.31–7.34 (m, 2H, ArH), 7.41 (dd, 1H, *J* = 8.4 Hz, 2.0 Hz, ArH), 7.95 (d, 1H, *J* = 2.0 Hz, ArH), 8.03 (d, 1H, *J* = 8.4 Hz, ArH), 8.19 (s, 1H, CH-triazole); ^13^C-NMR (*d*_6_-DMSO, 100 MHz) δ: 28.0, 52.5, 115.9 (d, 1C, *J* = 21.5 Hz), 121.2, 123.7, 124.6, 125.0, 130.6 (d, 1C, *J* = 8.3 Hz), 131.6, 132.7 (d, 1C, *J* = 3.1 Hz), 134.0, 143.0, 153.9, 161.1 (d, 1C, *J* = 243 Hz), 169.0; MS (ESI, *m*/*z*): 391.02 [M + H]^+^.

*2-(((1-Benzyl-1H-1,2,3-triazol-4-yl)methyl)thio)-5-chlorobenzo[d]thiazole* (**6r**) [29]. White solid, yield 75%, m.p. 142–144 °C. ^1^H-NMR (*d*_6_-DMSO, 400 MHz) δ: 4.68 (s, 2H, SCH_2_), 5.56 (s, 2H, NCH_2_), 7.25–7.30 (m, 5H, ArH), 7.41 (d, 1H, *J* = 8.4 Hz, ArH), 7.96 (d, 1H, *J* = 2.0 Hz, ArH), 8.03 (d, 1H, *J* = 8.4 Hz, ArH), 8.19 (s, 1H, CH-triazole); ^13^C-NMR (*d*_6_-DMSO, 100 MHz) δ: 28.0, 53.3, 121.2, 123.7, 124.7, 125.0, 128.3, 128.5, 129.1, 131.6, 134.0, 136.4, 142.9, 153.9, 169.0; MS (ESI, *m*/*z*): 373.03 [M + H]^+^.

*2-(((1-(4-(Tert-butyl)benzyl)-1H-1,2,3-triazol-4-yl)methyl)thio)-5-chlorobenzo[d]thiazole* (**6s**). Yellow solid, yield 78%, m.p. 94–95 °C. ^1^H-NMR (*d*_6_-DMSO, 400 MHz) δ: 1.22 (s, 9H, CH_3_), 4.67 (s, 2H, SCH_2_), 5.51 (s, 2H, NCH_2_), 7.15 (d, 2H, *J* = 8.4 Hz, ArH), 7.28 (d, 2H, *J* = 8.4 Hz, ArH), 7.40 (d, 1H, *J* = 8.4 Hz, ArH), 7.97 (d, 1H, *J* = 2.0 Hz, ArH), 8.03 (d, 1H, *J* = 8.4 Hz, ArH), 8.19 (s, 1H, CH-triazole); ^13^C-NMR (*d*_6_-DMSO, 100 MHz) δ: 28.0, 31.5, 34.7, 53.0, 121.2, 123.7, 124.7, 125.0, 125.9, 128.0, 131.6, 133.5, 134.0, 142.9, 151.0, 153.9, 169.0; MS (ESI, *m*/*z*): 429.09 [M + H]^+^.

### 3.6. In Vitro Assay of α-Glucosidase Inhibitory Activity

α-Glucosidase inhibitory activity was assayed by using 0.1 M phosphate buffer (pH 6.8) at 37 °C. The enzyme (0.1 U/mL) in phosphate buffer saline was incubated with various concentrations of test compounds at 37 °C for 15 min. Then 1.25 mM *p*-nitrophenyl α-d-glucopyranoside was added to the mixture as a substrate. After further incubation at 37 °C for 30 min. The absorbance was measured spectrophotometrically at 405 nm. The sample solution was replaced by DMSO as a control. Acarbose was used as a positive control.

### 3.7. Molecular Docking

Molecular docking studies were performed to investigate the binding mode between **6i**, **6s** and α-glucosidase using Autodock vina 1.1.2. The 3D structure of α-glucosidase of *Saccharomyces cerevisiae* have been predicted using homology modeling in our previous report [25]. The 3D structure of the compounds were obtained by ChemBioDraw Ultra 14.0 and ChemBio3D Ultra 14.0 softwares. The AutoDockTools 1.5.6 package was employed to generate the docking input files. The search grid of α-glucosidase was identified as center_x: −19.676, center_y: −7.243, and center_z: −21.469, with dimensions size_x: 15, size_y: 15, and size_z: 15. The value of exhaustiveness was set to 20. For Vina docking, the default parameters were used if it was not mentioned. The best-scoring pose as judged by the Vina docking score was chosen and visually analyzed using PyMoL 1.7.6 software (http://www.pymol.org/).

## 4. Conclusions

In summary, a novel series of benzothiazole-triazole derivatives **6a**–**6s** have been synthesized and evaluated for their α-glucosidase inhibitory activity. The majority of compounds exhibited superior α-glucosidase inhibitory activity in the range of IC_50_ = 20.7 and 61.1 μM as compared to standard acarbose (IC_50_ = 817.38 μM). Molecular docking study was carried out to understand the molecular interaction of compounds with the active site of α-glucosidase. This study has identified a novel series of lead compounds which can be used for the development of new α-glucosidase inhibitors.

## Figures and Tables

**Table 1:** α-Glucosidase inhibitory activity of novel benzothiazole-triazole derivatives (**6a**–**6s**).

Compound	R_1_	R_2_	IC_50_ (μM)
**6a**	H	4-Br	28.7
**6b**	H	2-Cl	>100
**6c**	H	2-Br	37.4
**6d**	H	3-F	61.1
**6e**	H	4-Cl	27.4
**6f**	H	2-F	>100
**6g**	H	H	>100
**6h**	H	3,5-Cl_2_	41.0
**6i**	H	4-tBu	29.4
**6j**	Cl	2-F	45.9
**6k**	Cl	2-Br	48.4
**6l**	Cl	2-Cl	33.6
**6m**	Cl	4-Cl	28.2
**6n**	Cl	4-Br	28.0
**6o**	Cl	3,5-Cl_2_	22.3
**6p**	Cl	3-F	38.6
**6q**	Cl	4-F	53.1
**6r**	Cl	H	44.4
**6s**	Cl	4-tBu	20.7
Acarbose			817.38

## Supplementary Materials

Supplementary Materials are available online.
