# Supplementary material for: Synthesis, In Vitro α-Glucosidase Inhibitory Activity and Molecular Docking Studies of Novel Benzothiazole-Triazole Derivatives
Source: Molecules. 2017 Sep 15;22(9):1555. doi: 10.3390/molecules22091555 (PMC6151782; doi:10.3390/molecules22091555)
Supplement: Supplementary file 1 [file molecules-22-01555-s001.pdf]

Supplemental Material

Synthesis, *in vitro* α-glucosidase inhibitory activity and molecular docking studies of novel benzothiazole-triazole derivatives

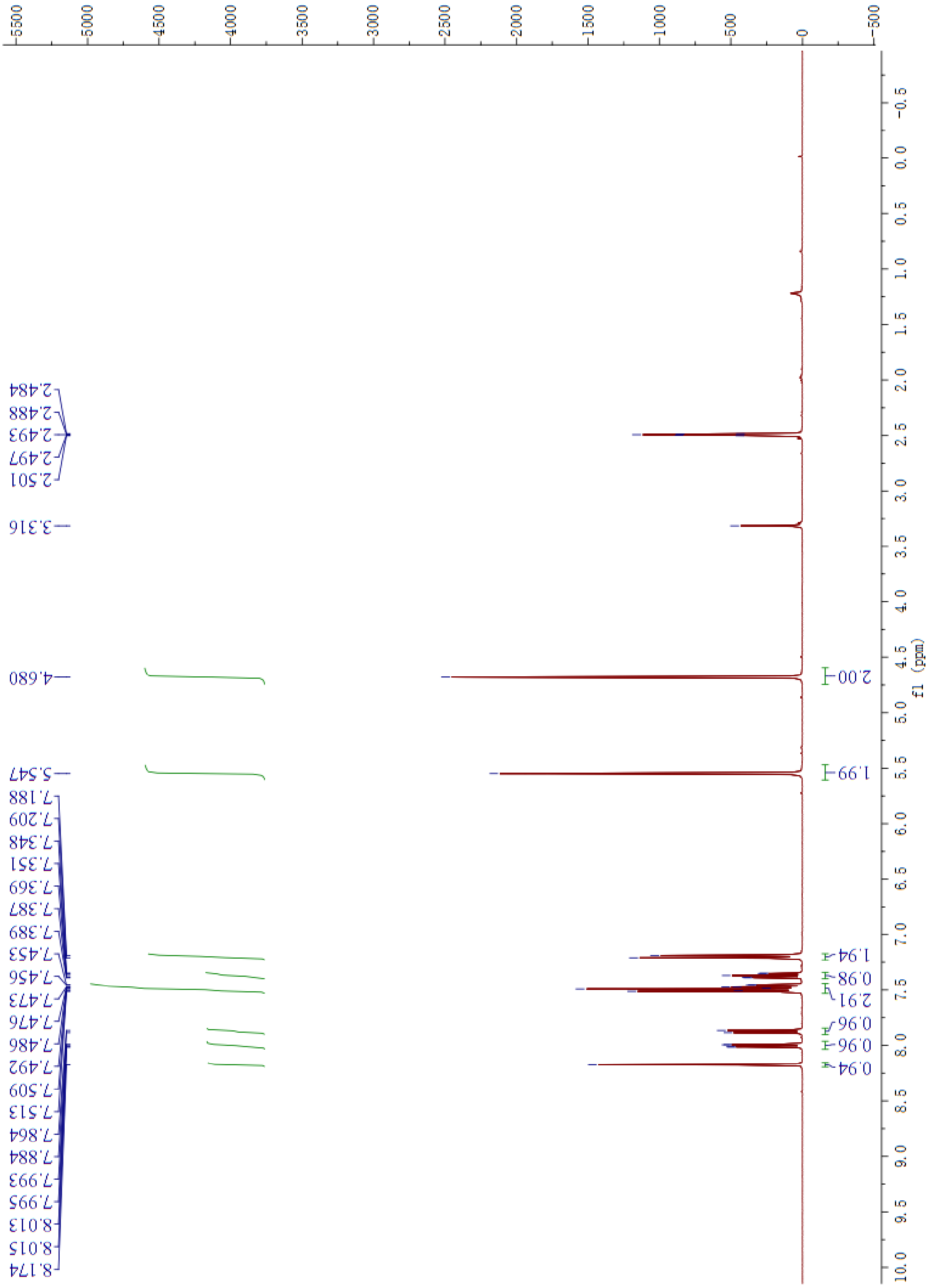

Figure S 1: ¹H NMR of compound 6a

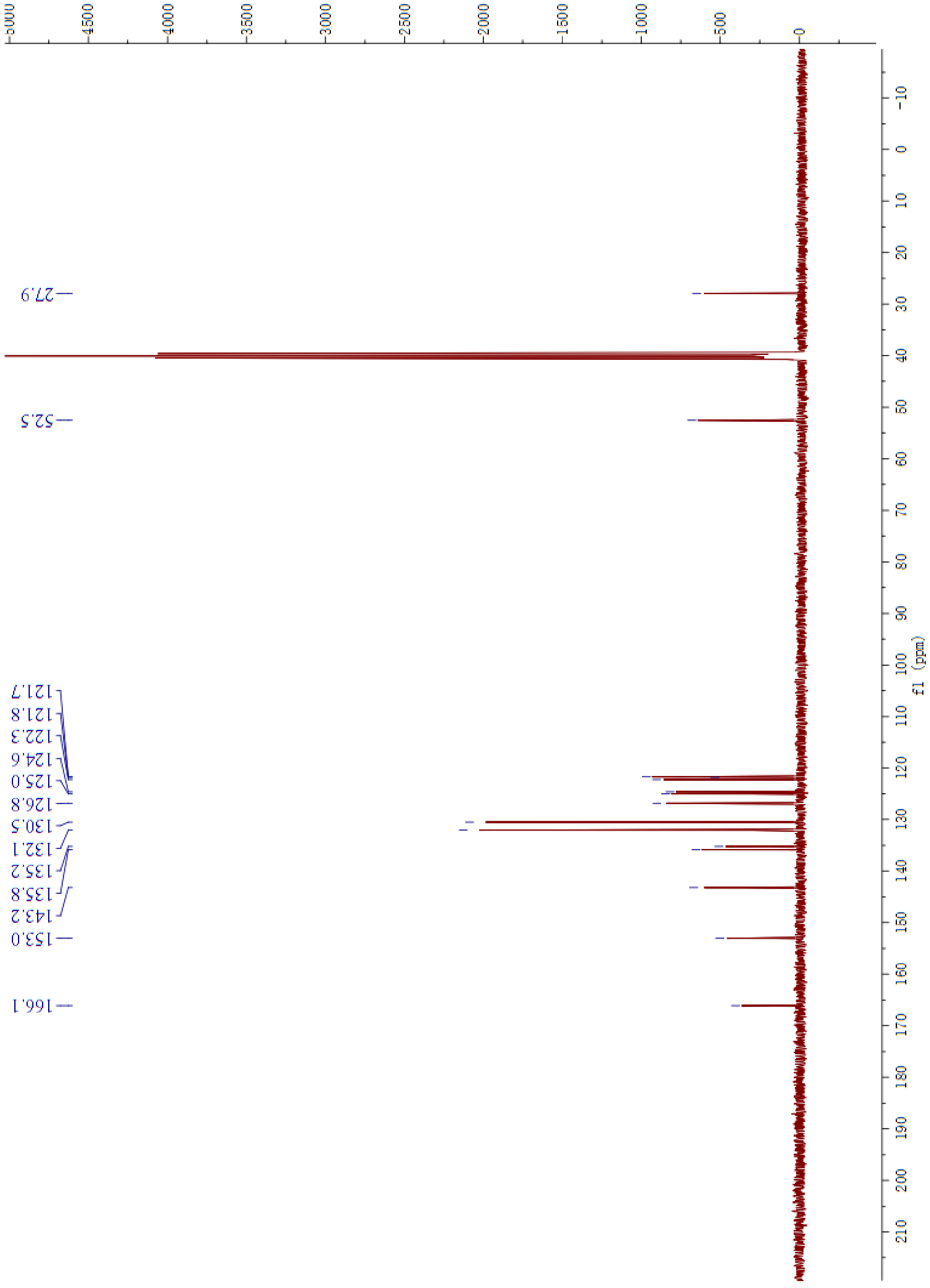

Figure S 2: ^{13}C NMR of compound 6a

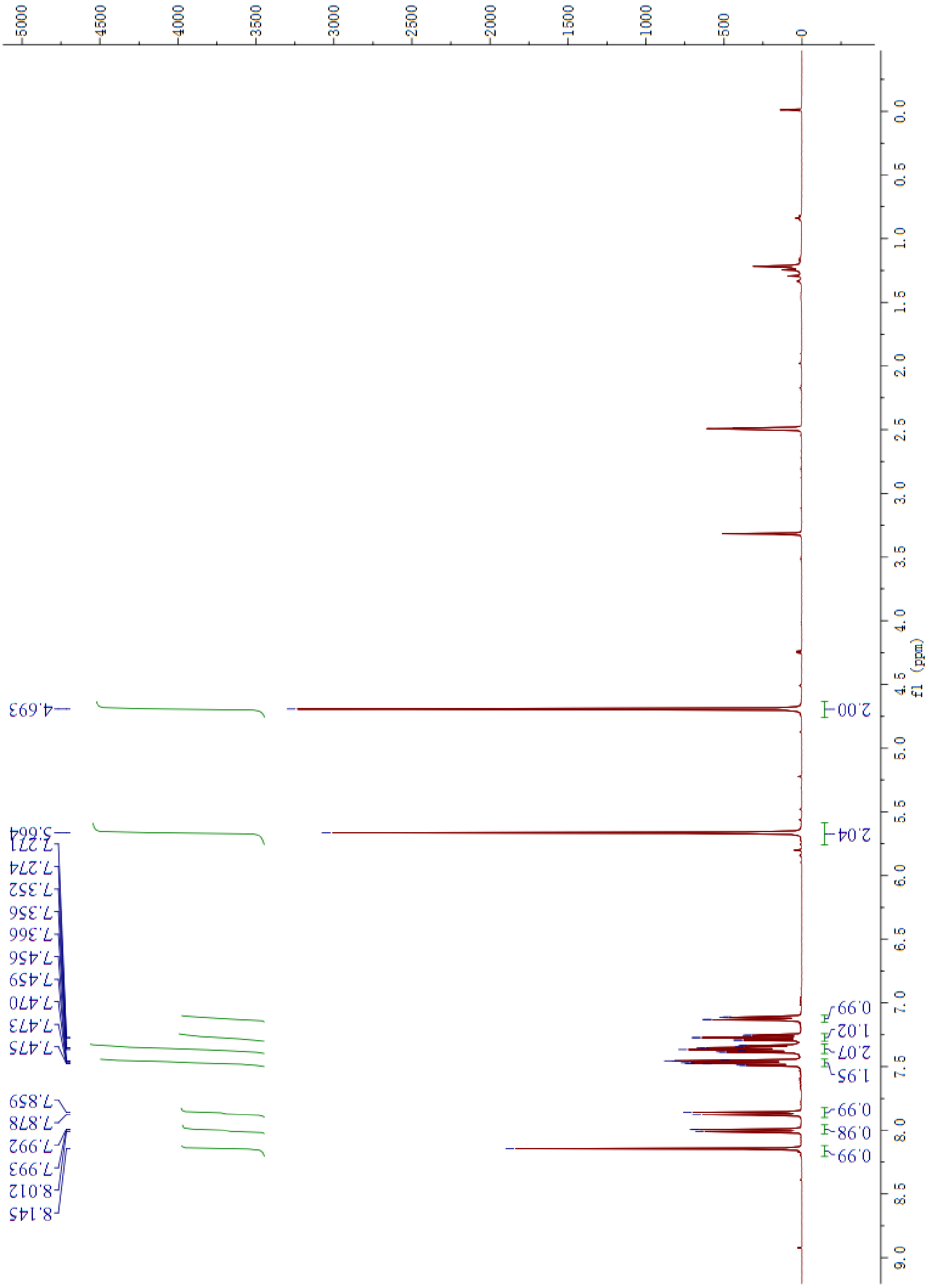

Figure S 3: ^{1}H NMR of compound 6b

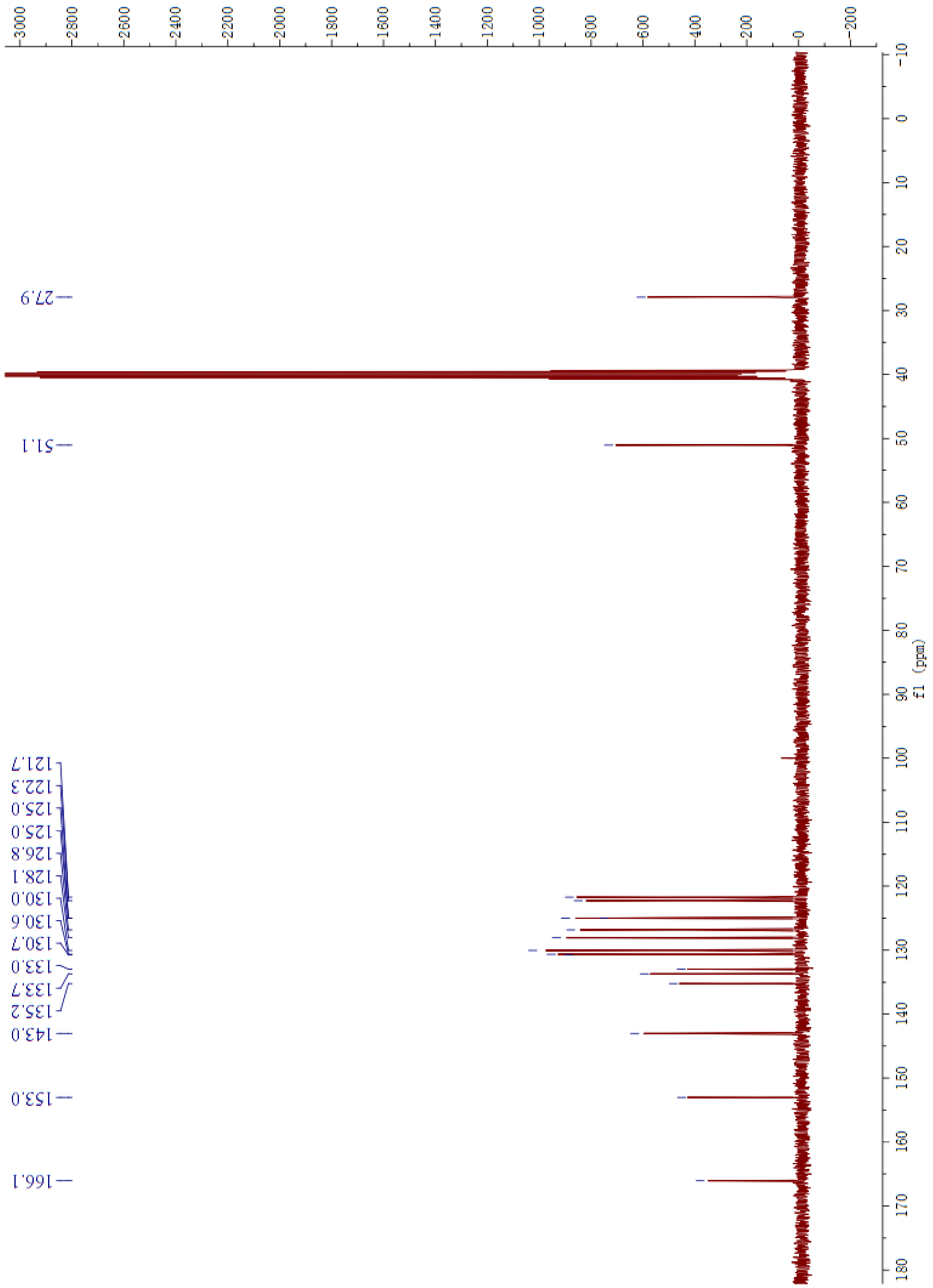

Figure S 4: ^{13}C NMR of compound 6b

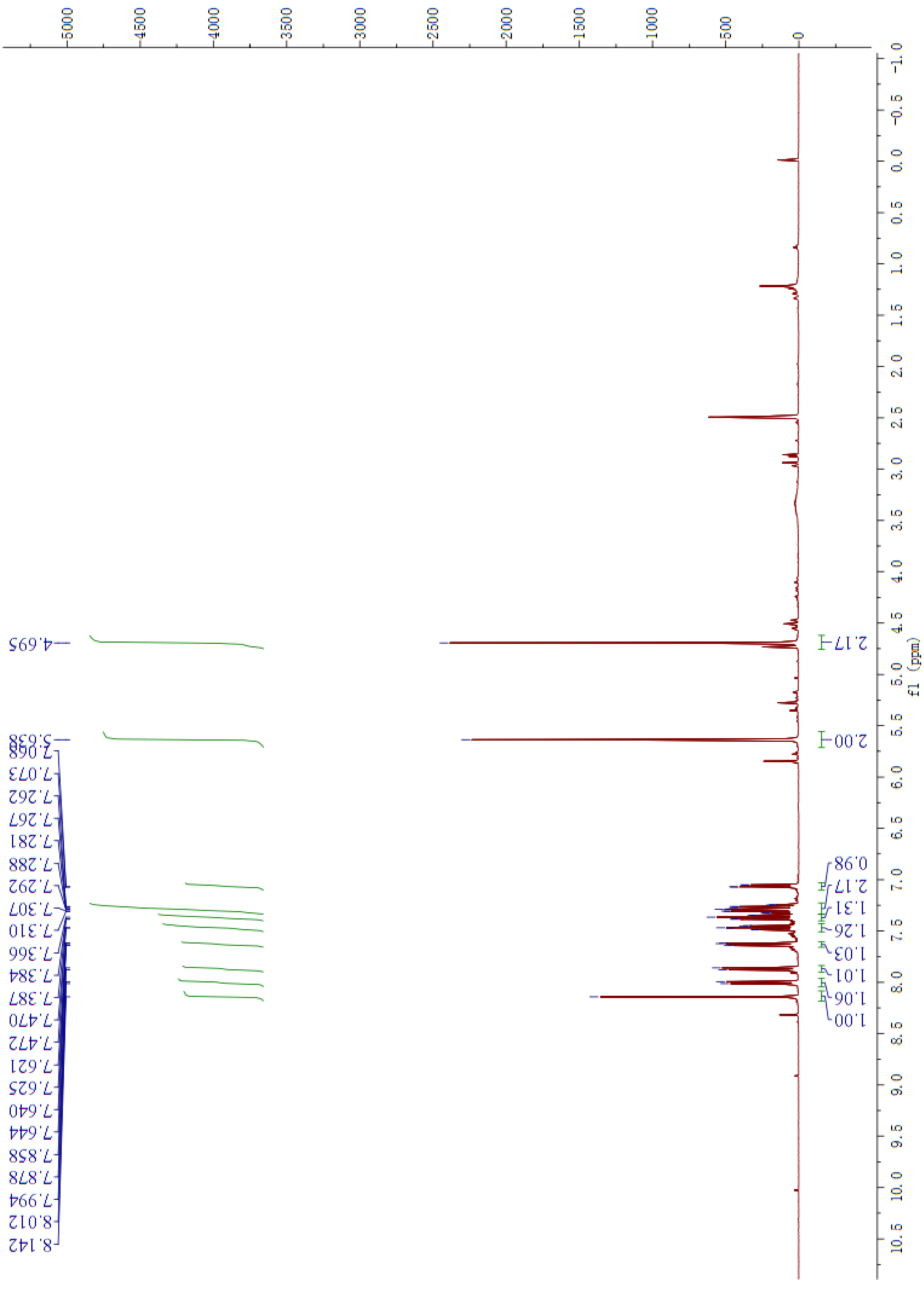

Figure S 5: ^{1}H NMR of compound 6c

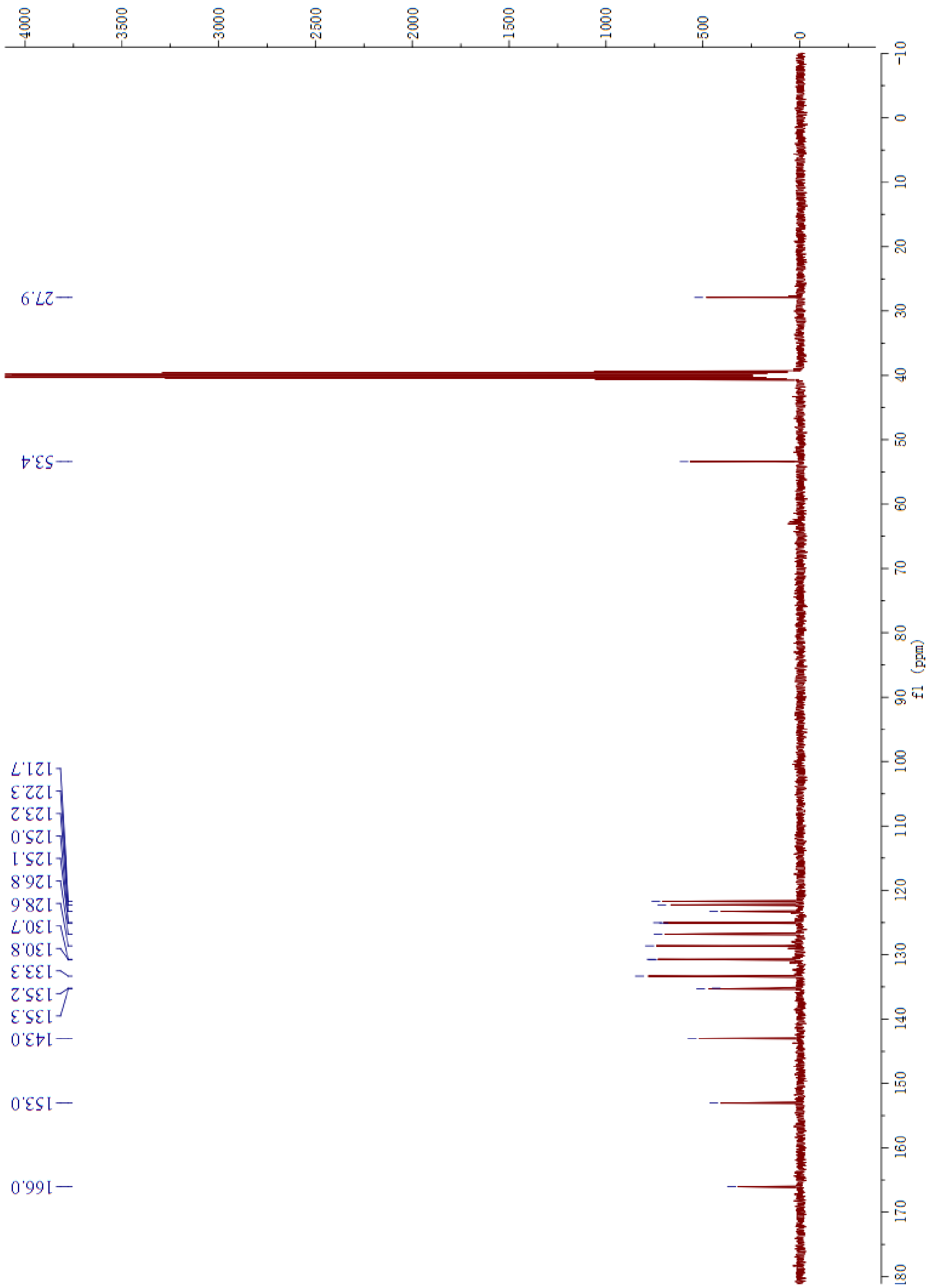

Figure S 6: ^{13}C NMR of compound 6c

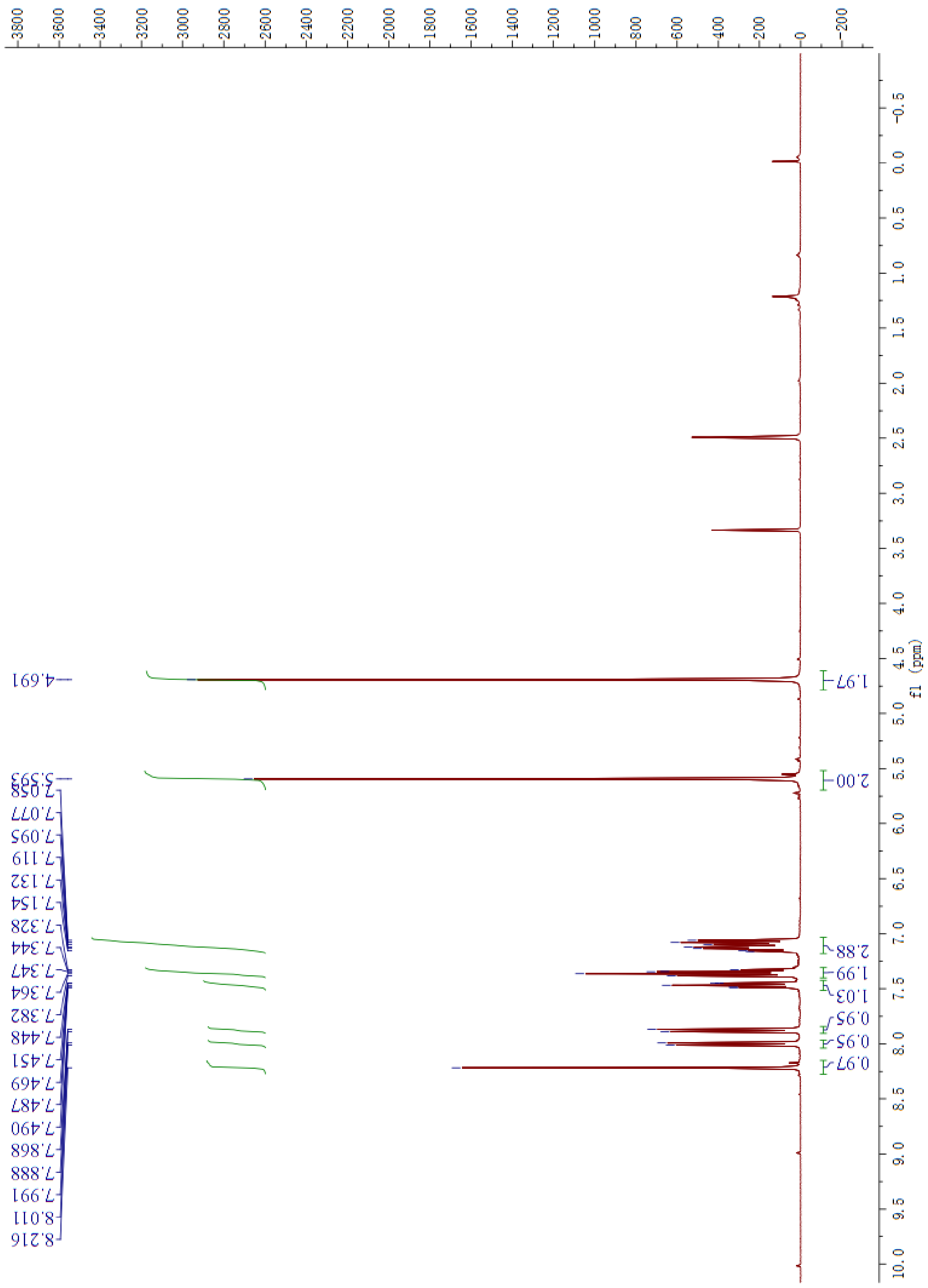

Figure S 7: ^{1}H NMR of compound 6d

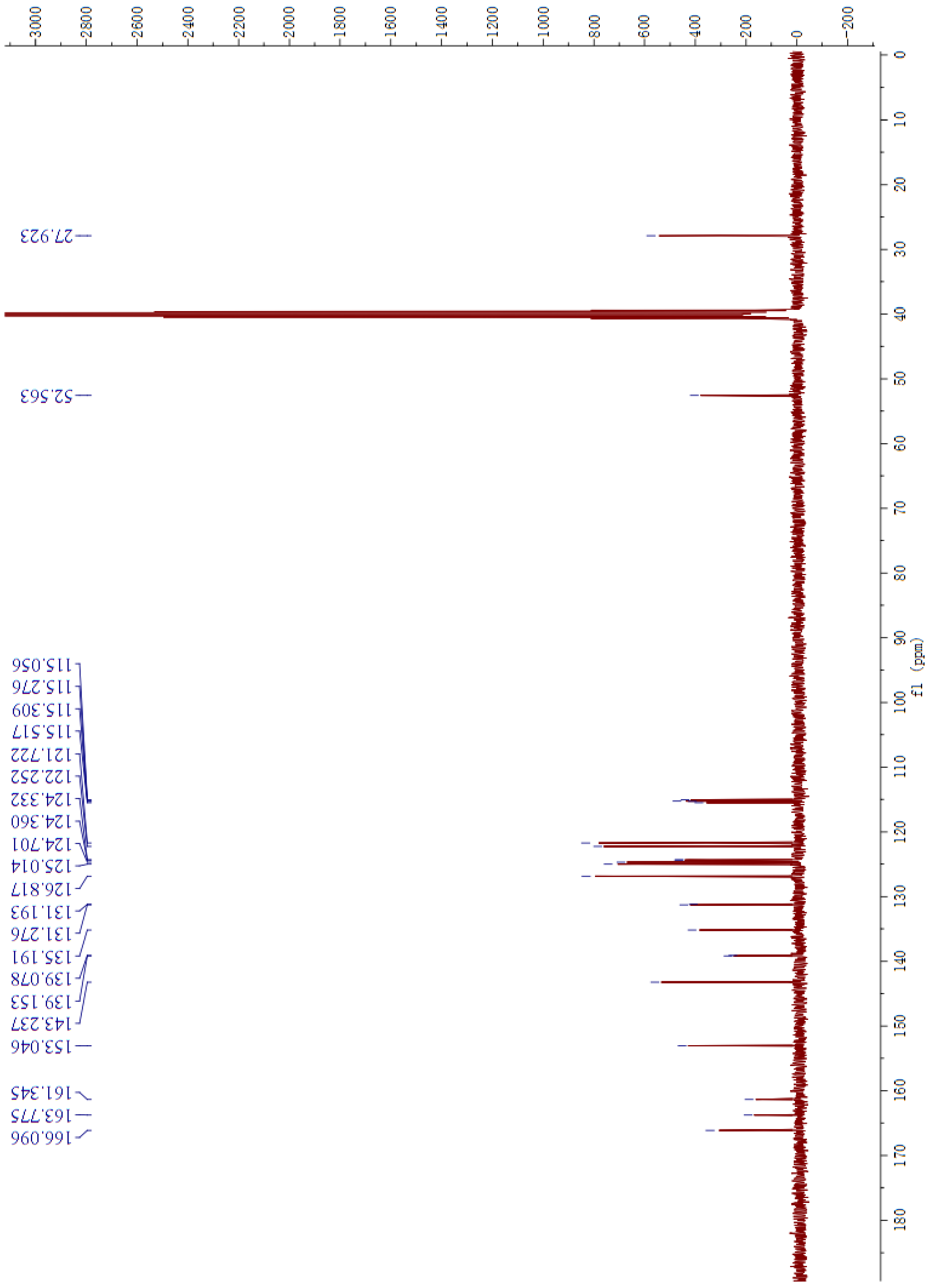

Figure S 8: ^{13}C NMR of compound 6d

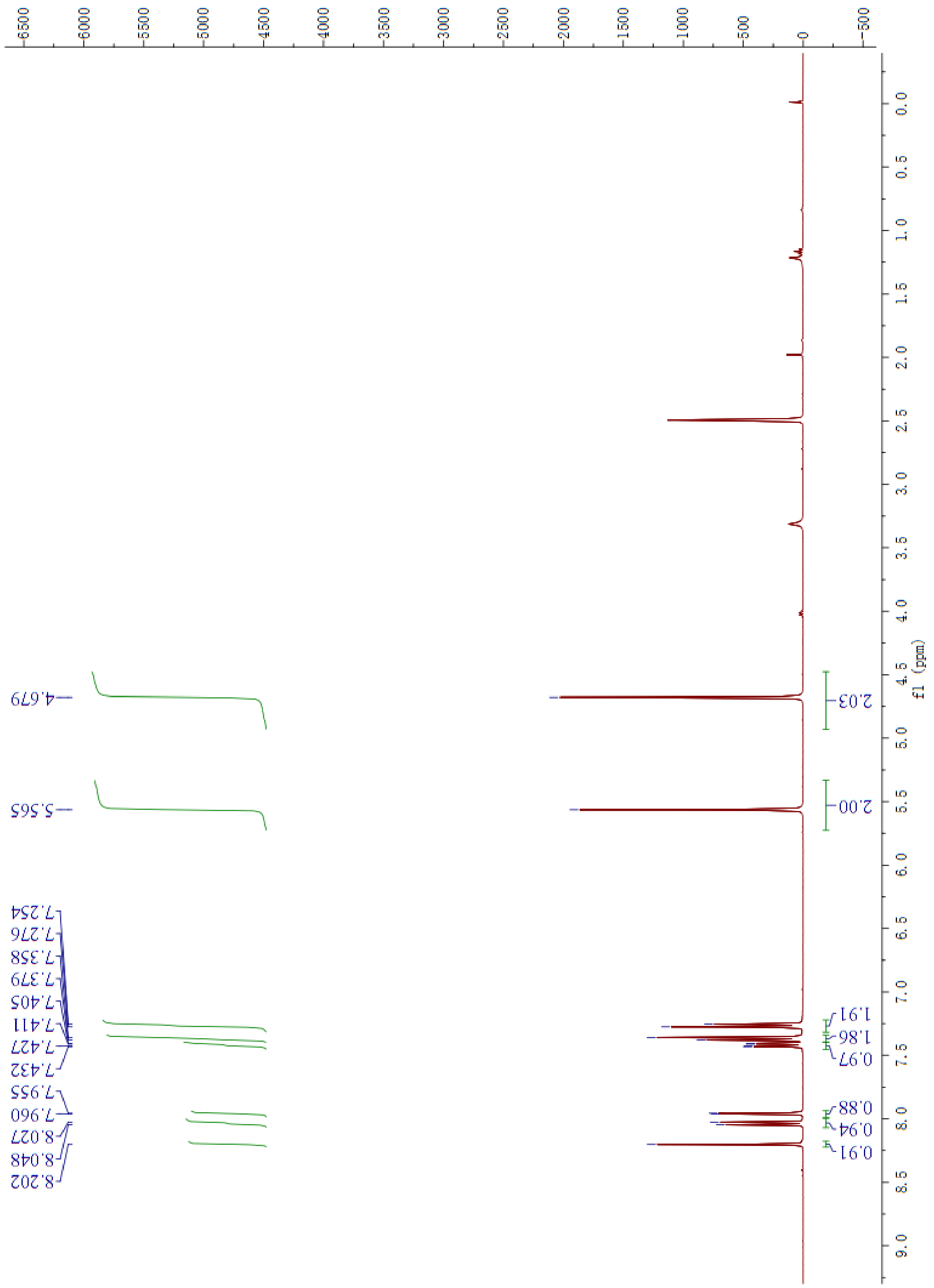

Figure S 9: ^{1}H NMR of compound 6e

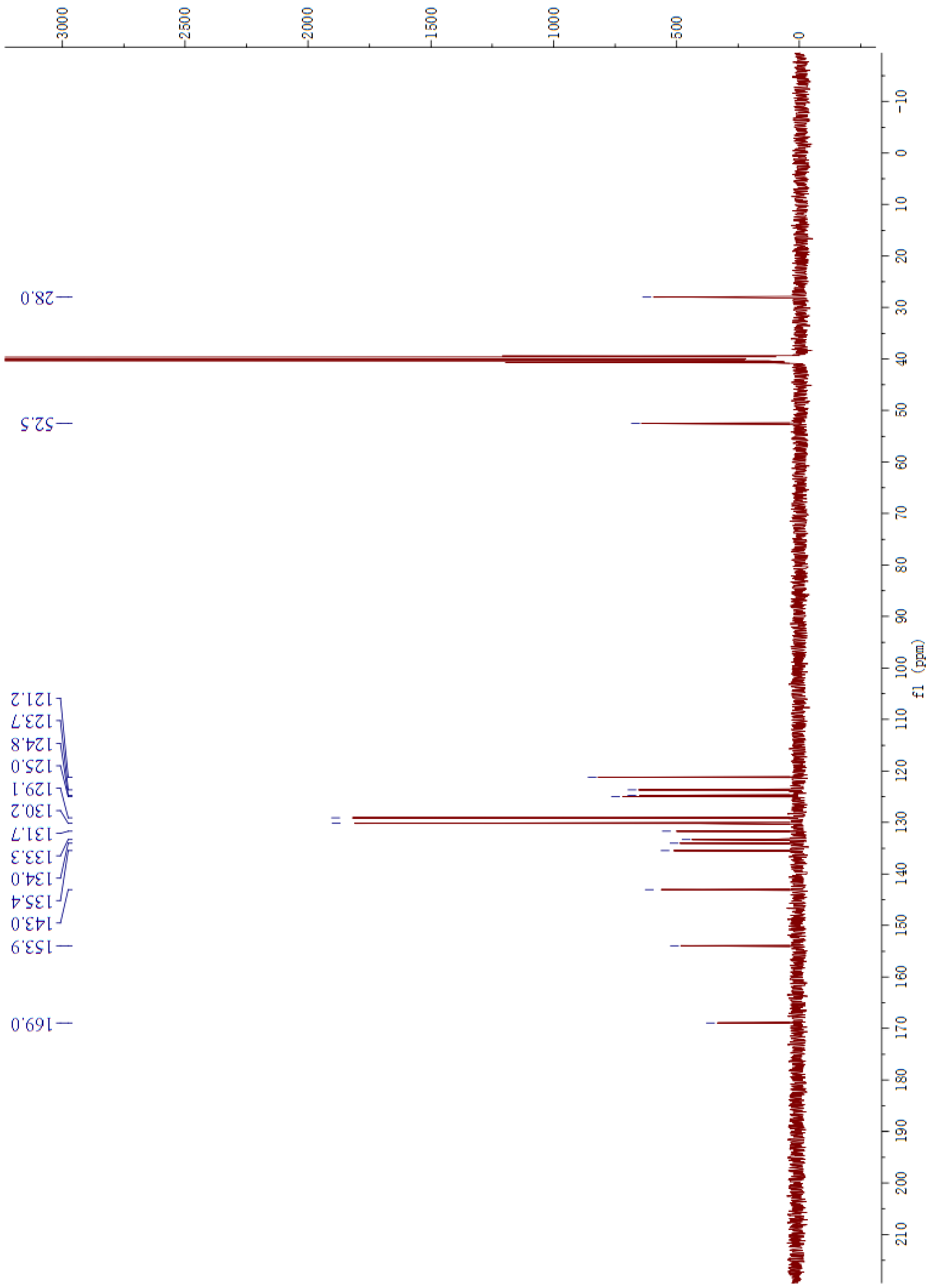

Figure S 10: ^{13}C NMR of compound 6e

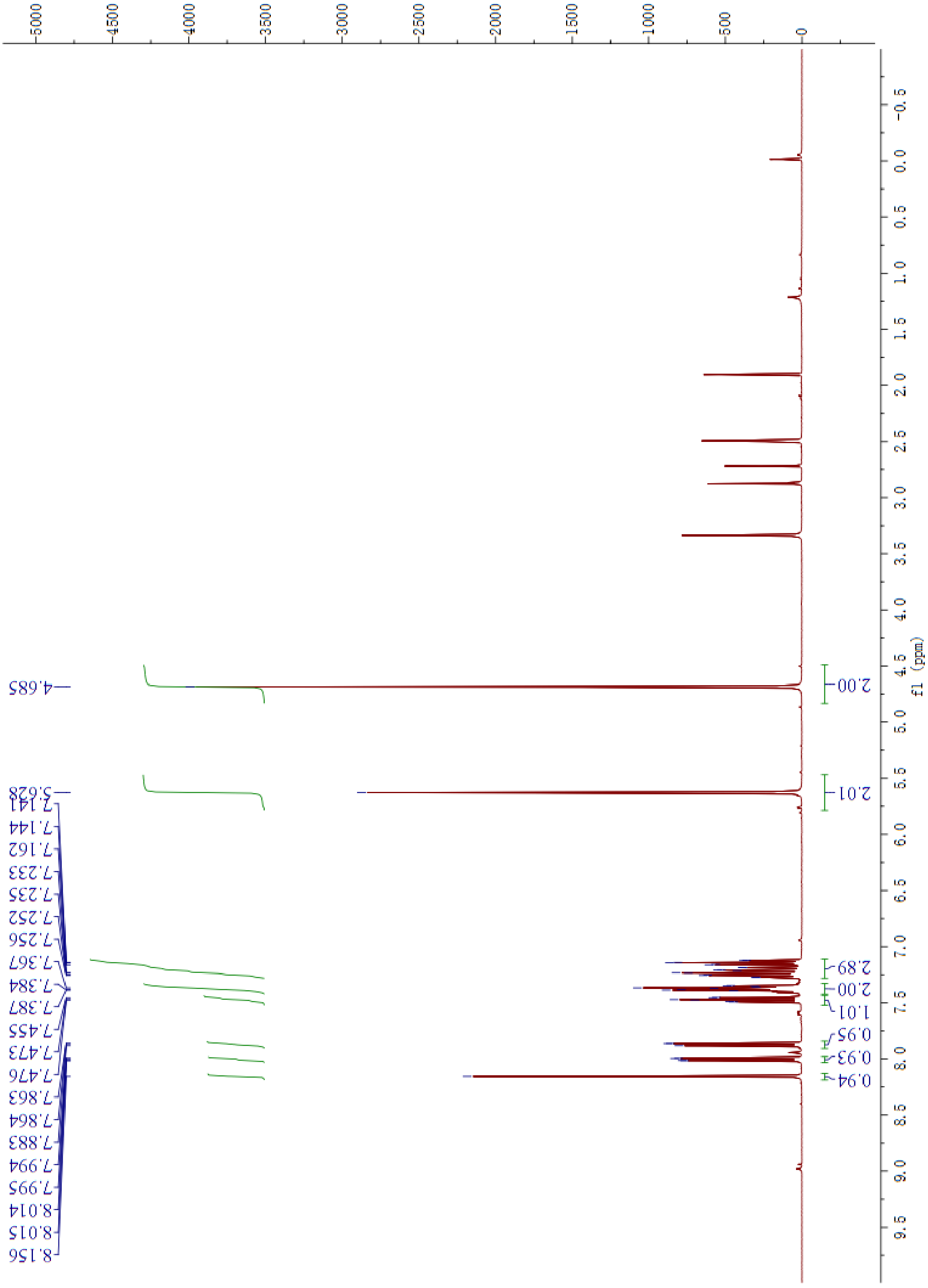

Figure S 11: ^{1}H NMR of compound 6f

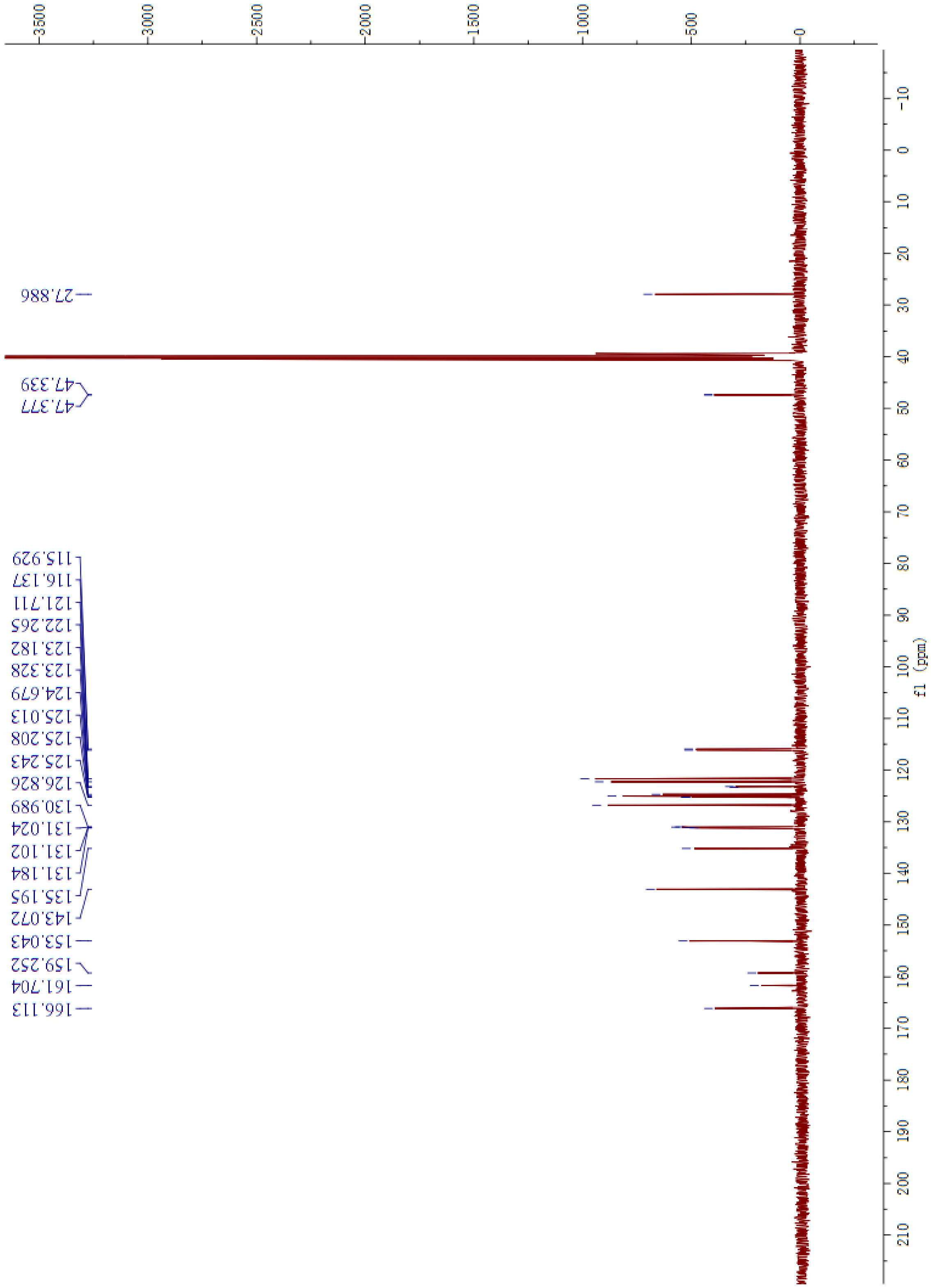

Figure S 12: ^{13}C NMR of compound 6f

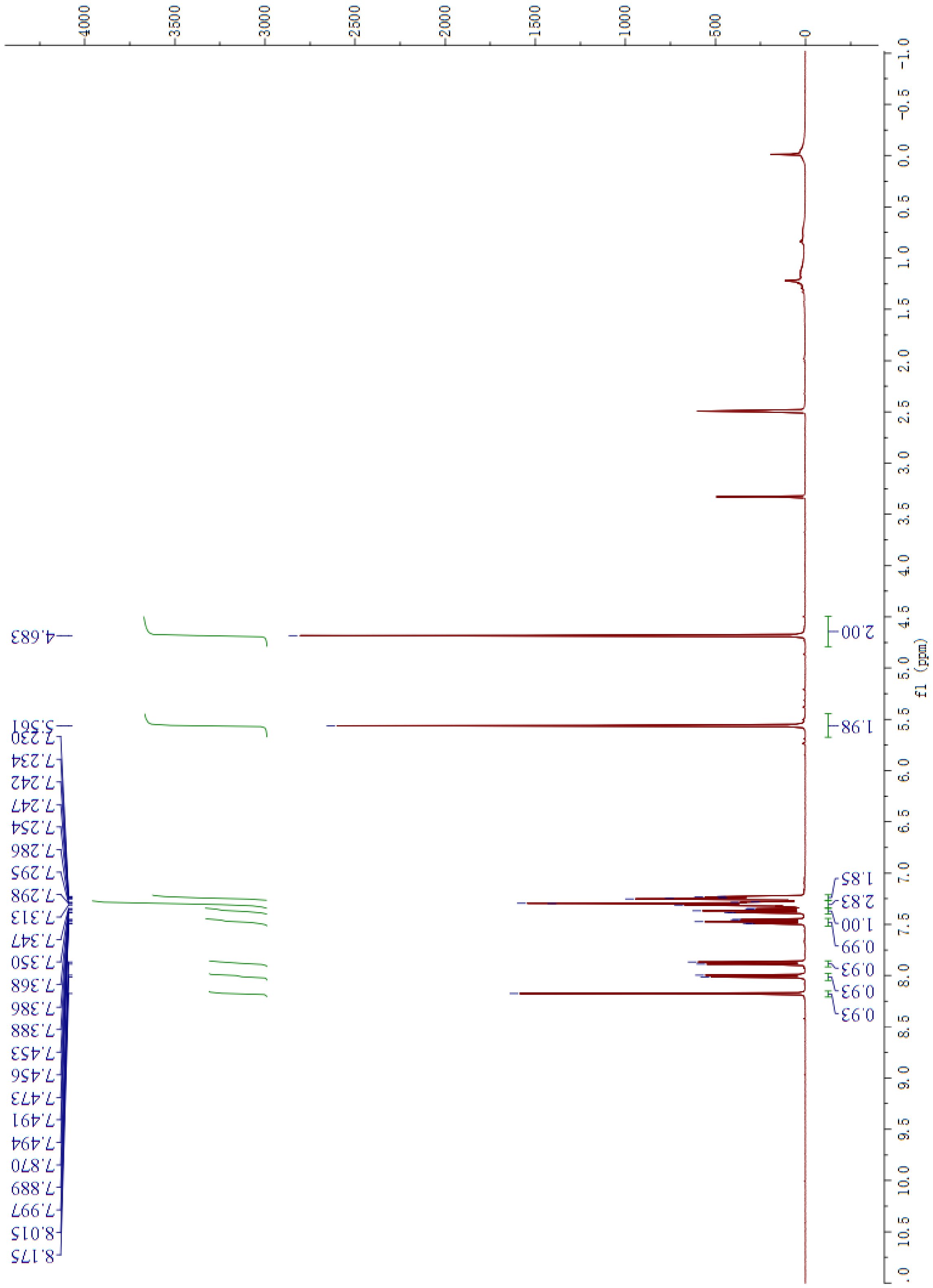

Figure S 13: ^{1}H NMR of compound 6g

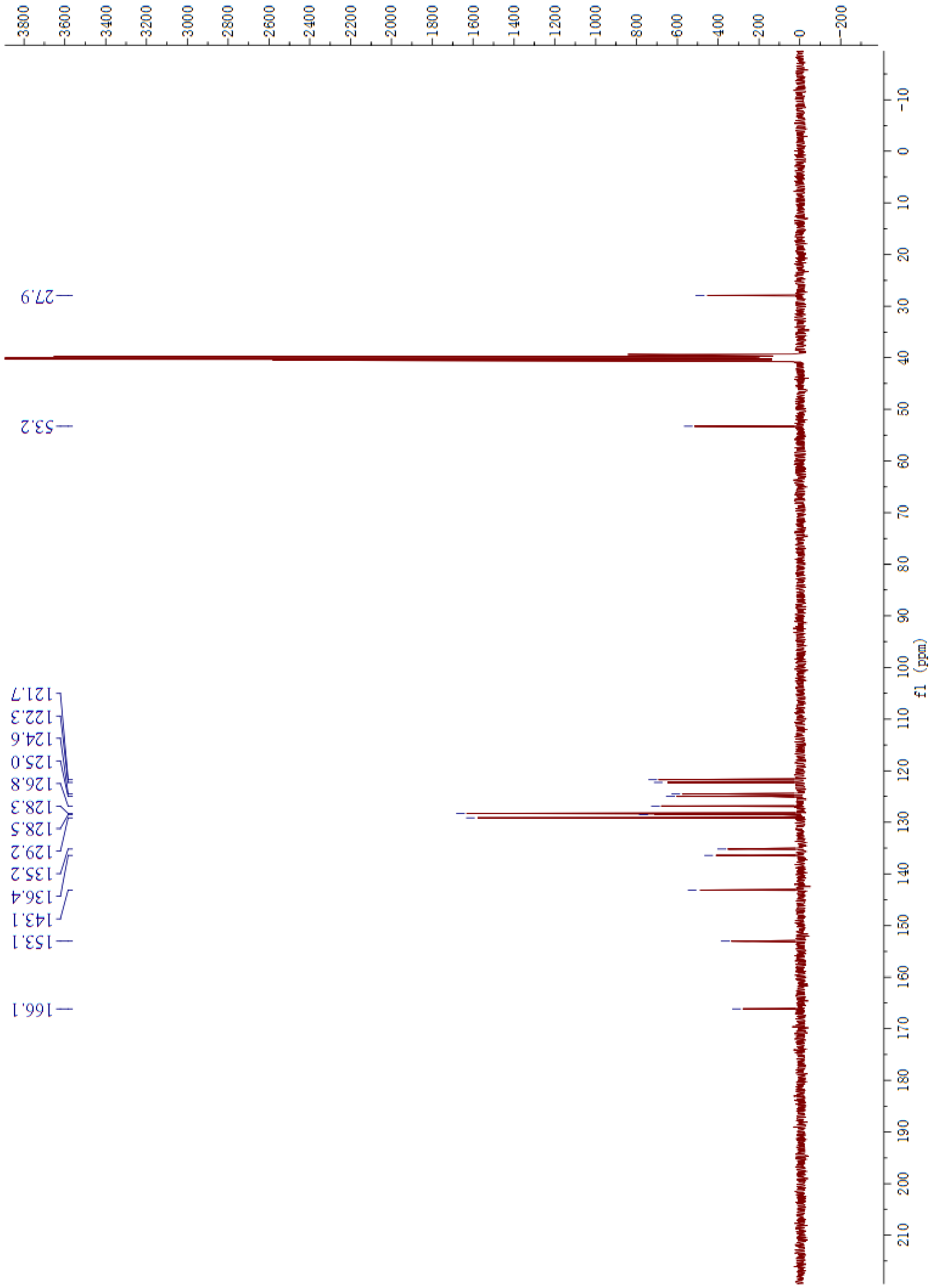

Figure S 14: ^{13}C NMR of compound 6g

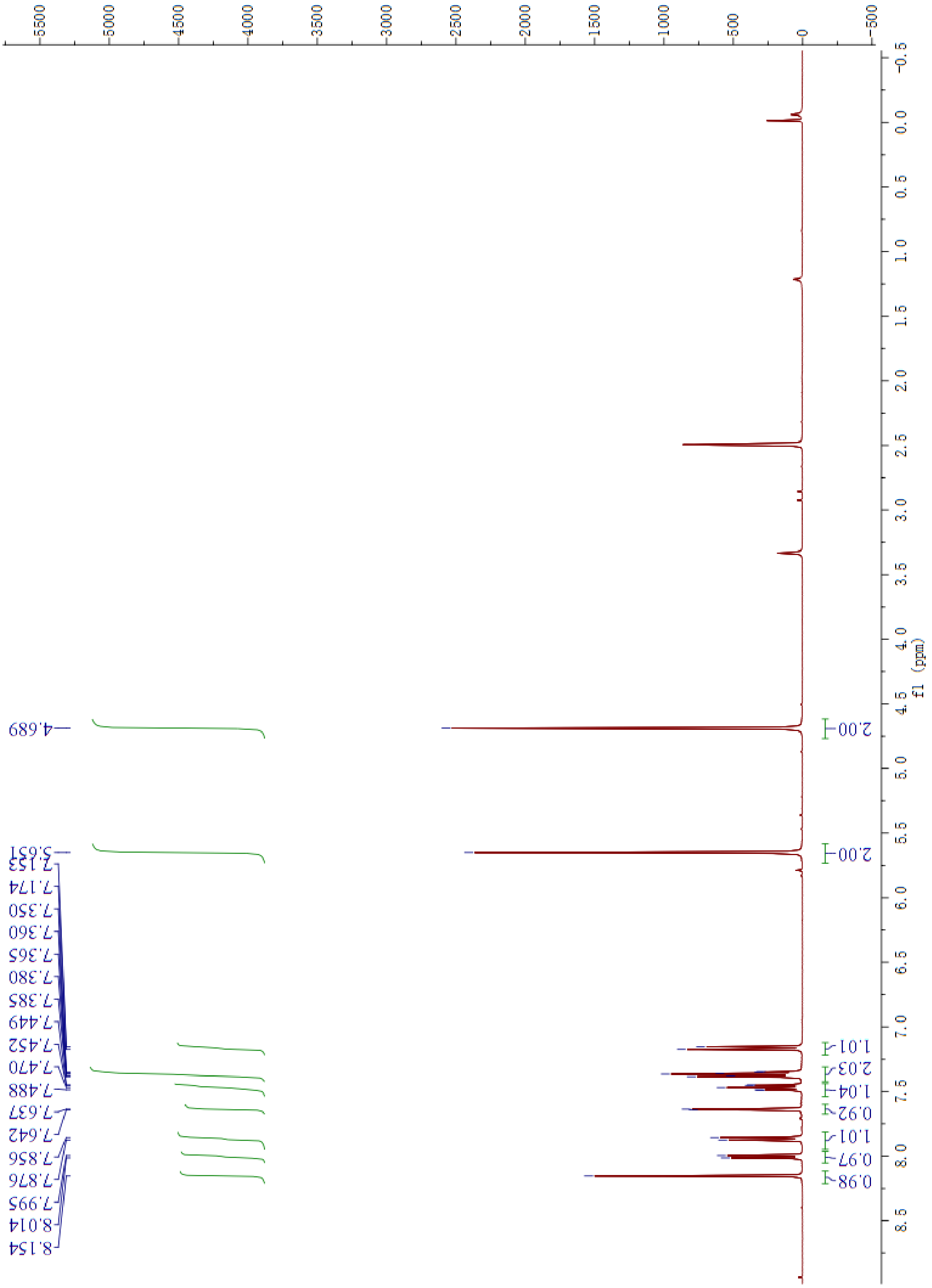

Figure S 15: ^{1}H NMR of compound 6h

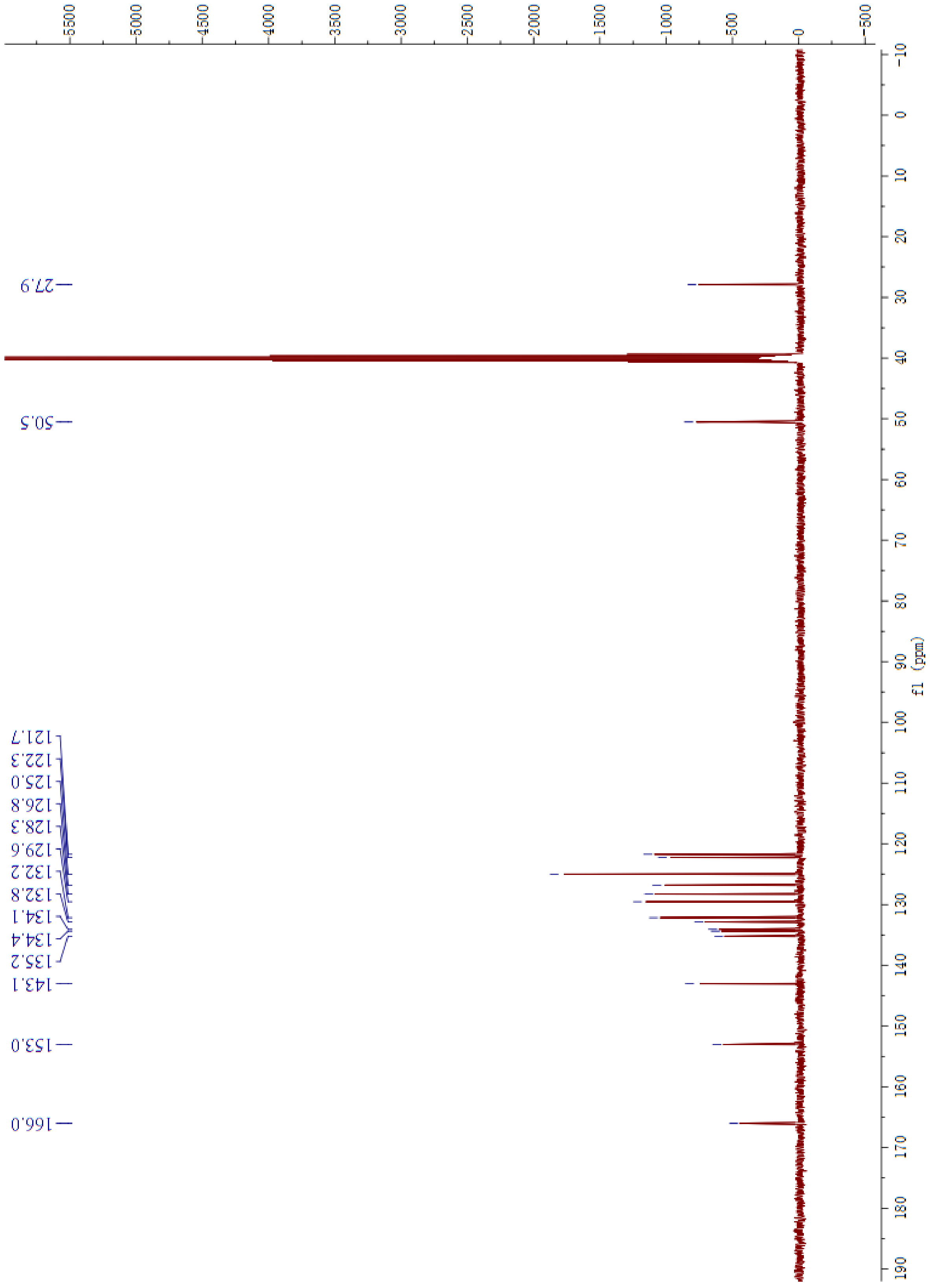

Figure S 16: ^{13}C NMR of compound 6h

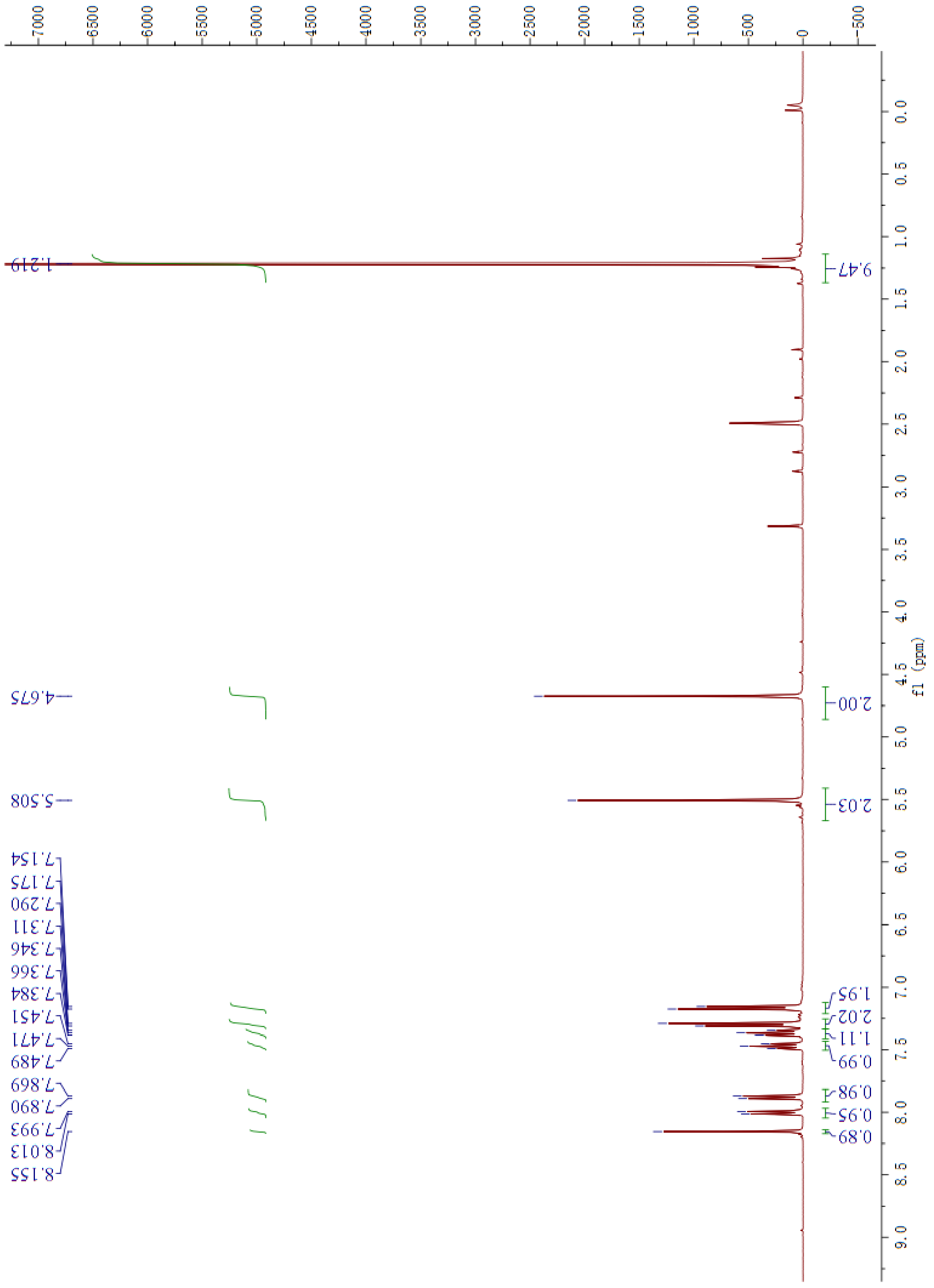

Figure S 17: ^{1}H NMR of compound 6i

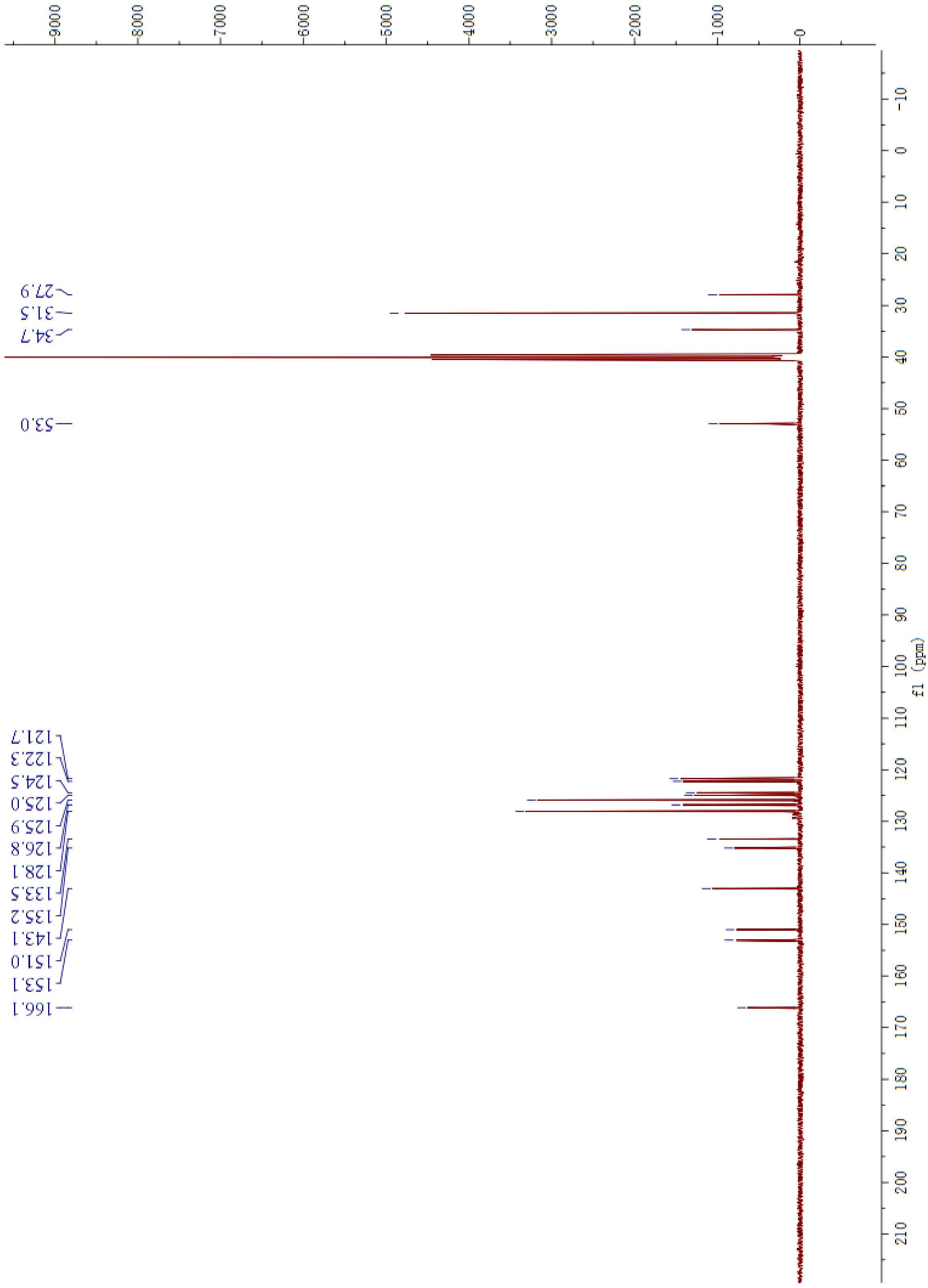

Figure S 18: ^{13}C NMR of compound 6i

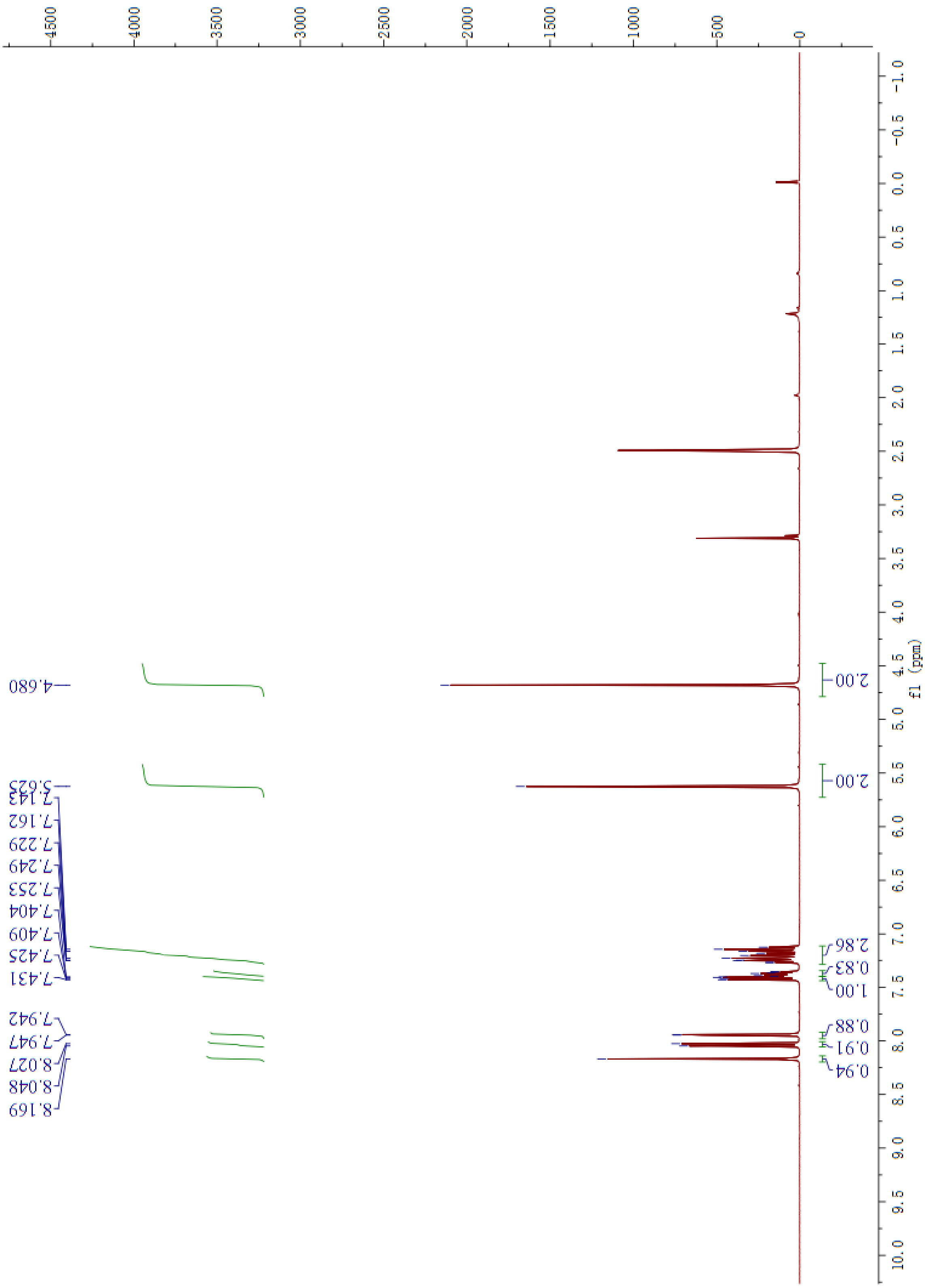

Figure S 19: ¹H NMR of compound 6j

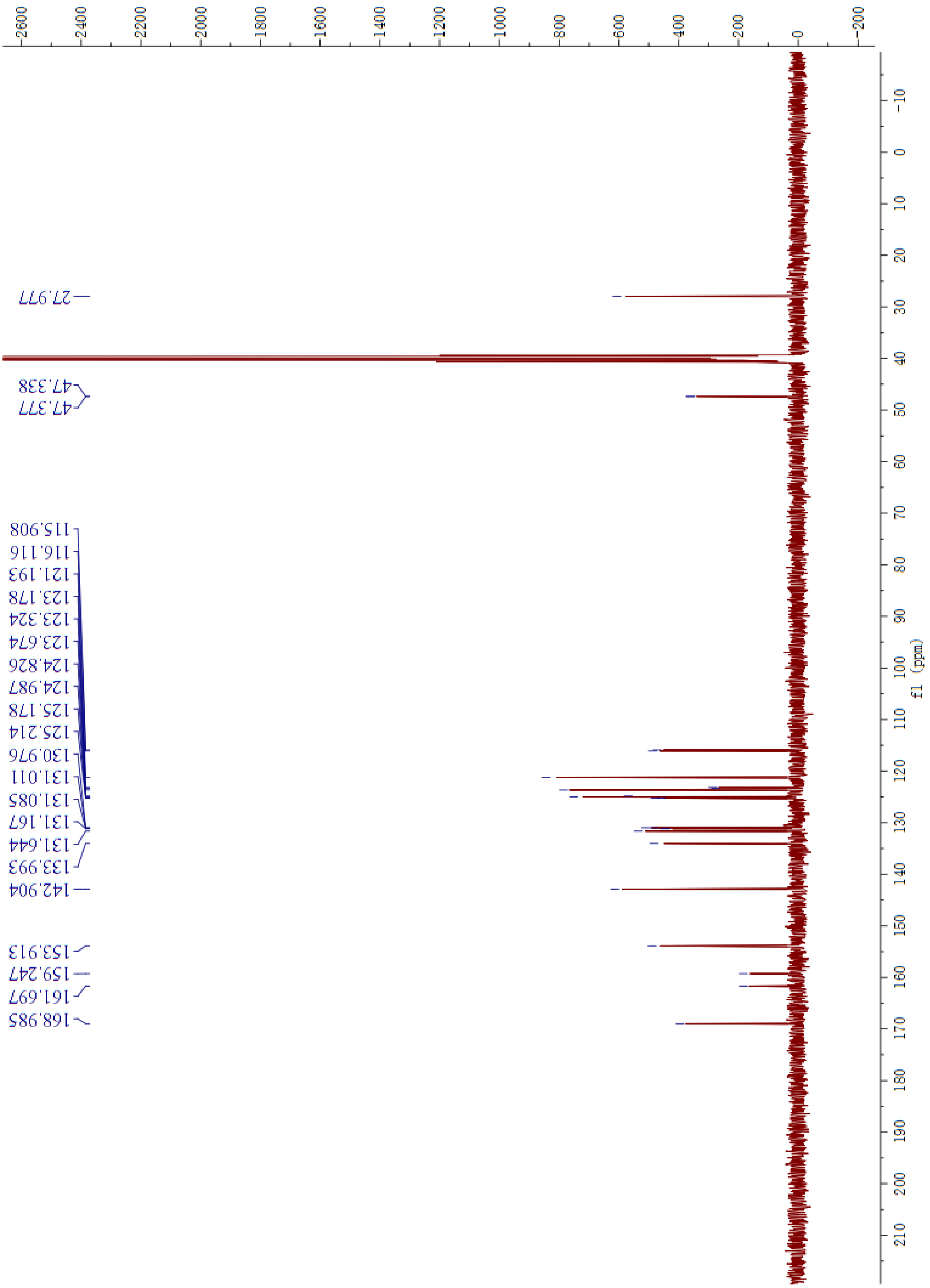

Figure S 20: ^{13}C NMR of compound 6j

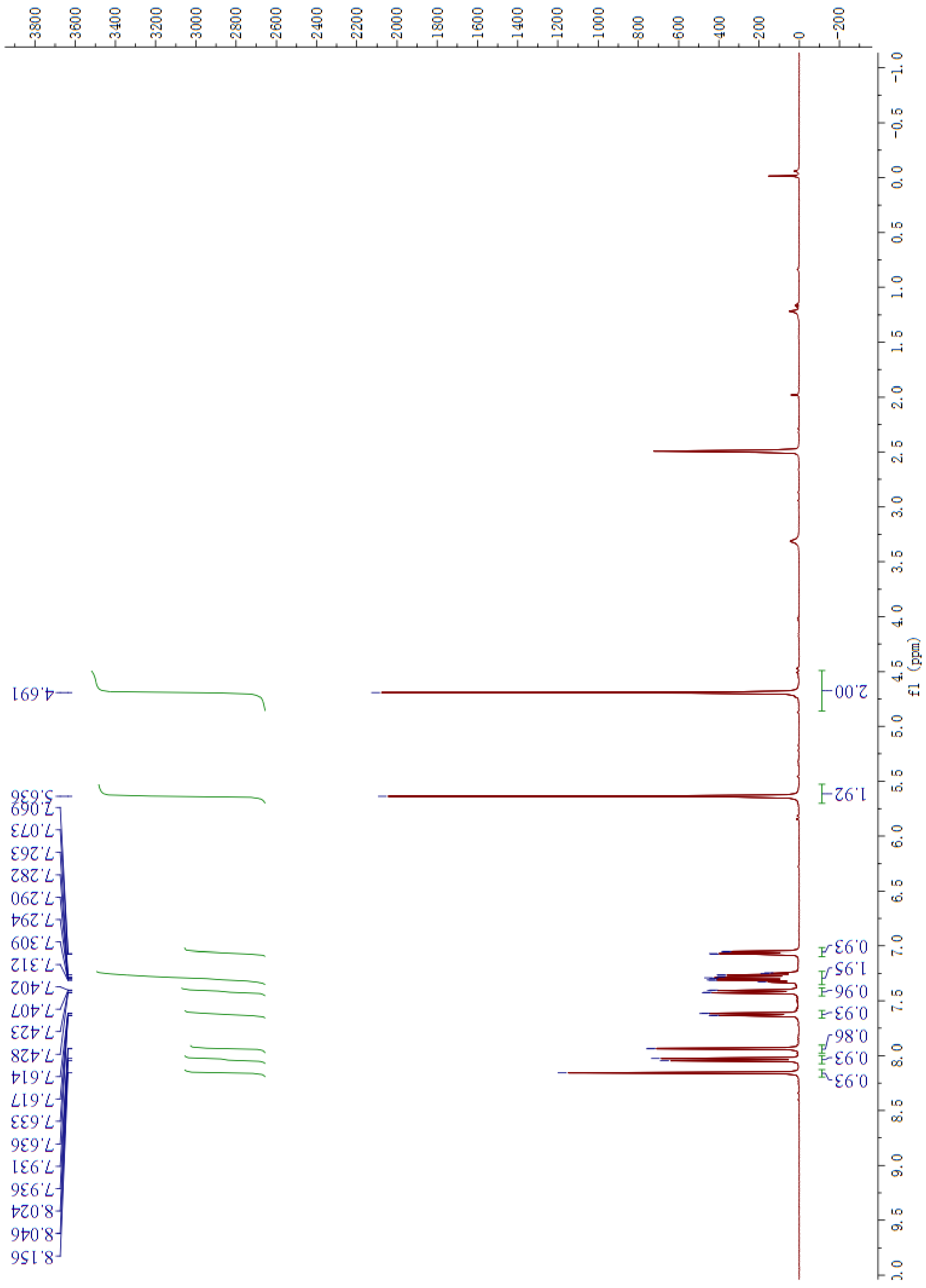

Figure S 21: ^{1}H NMR of compound 6k

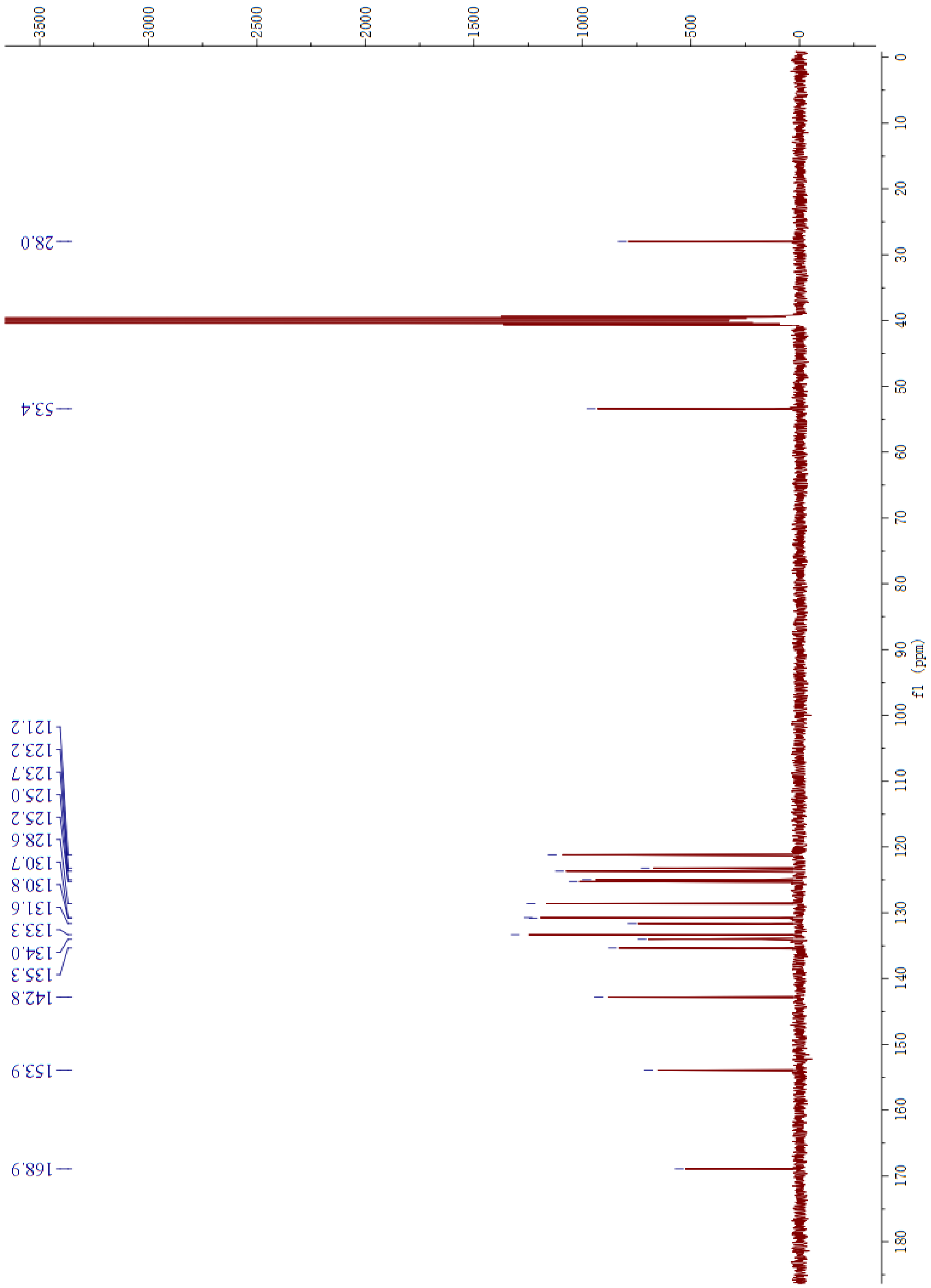

Figure S 22: ^{13}C NMR of compound 6k

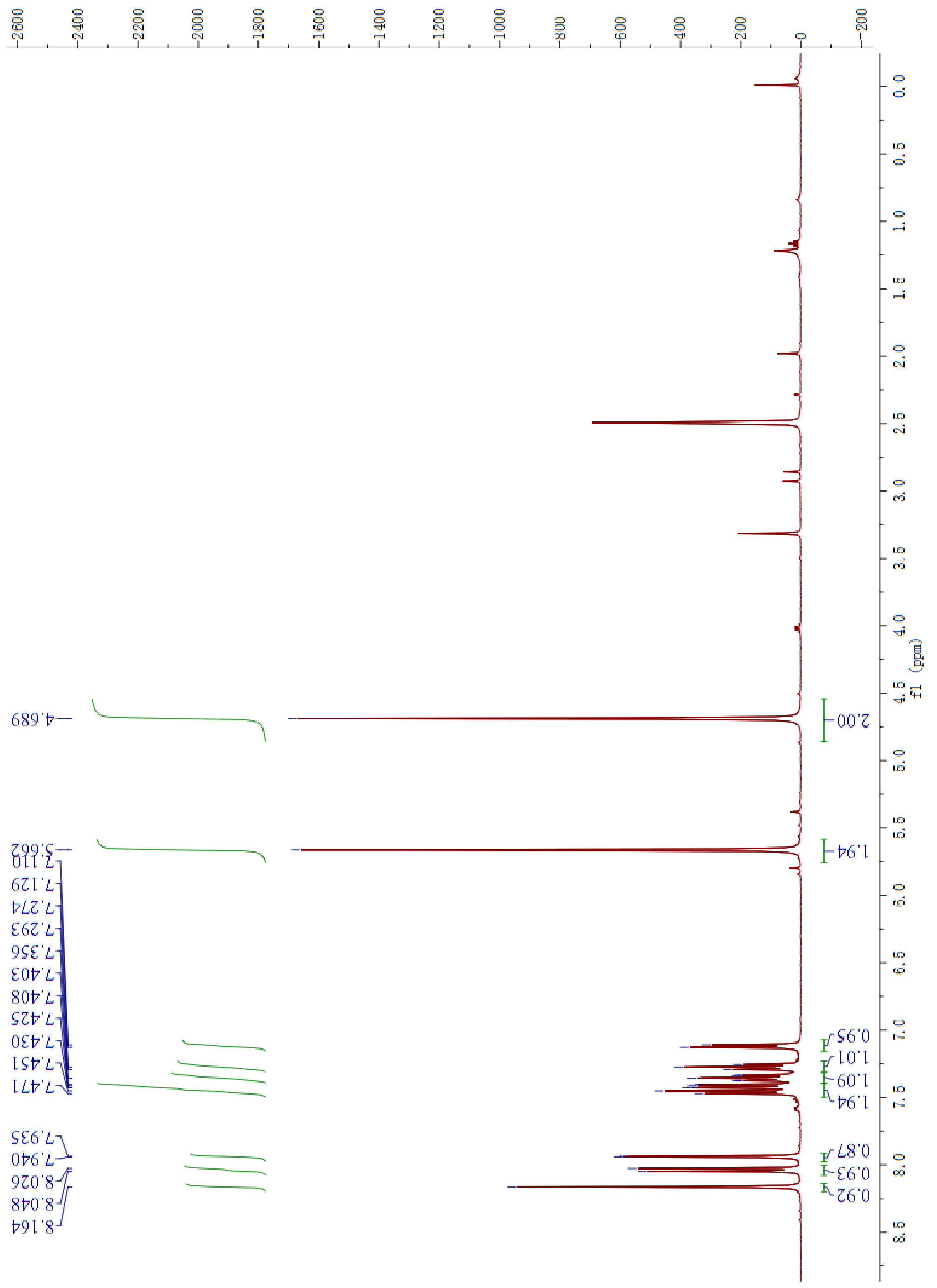

Figure S 23: ^{1}H NMR of compound 6l

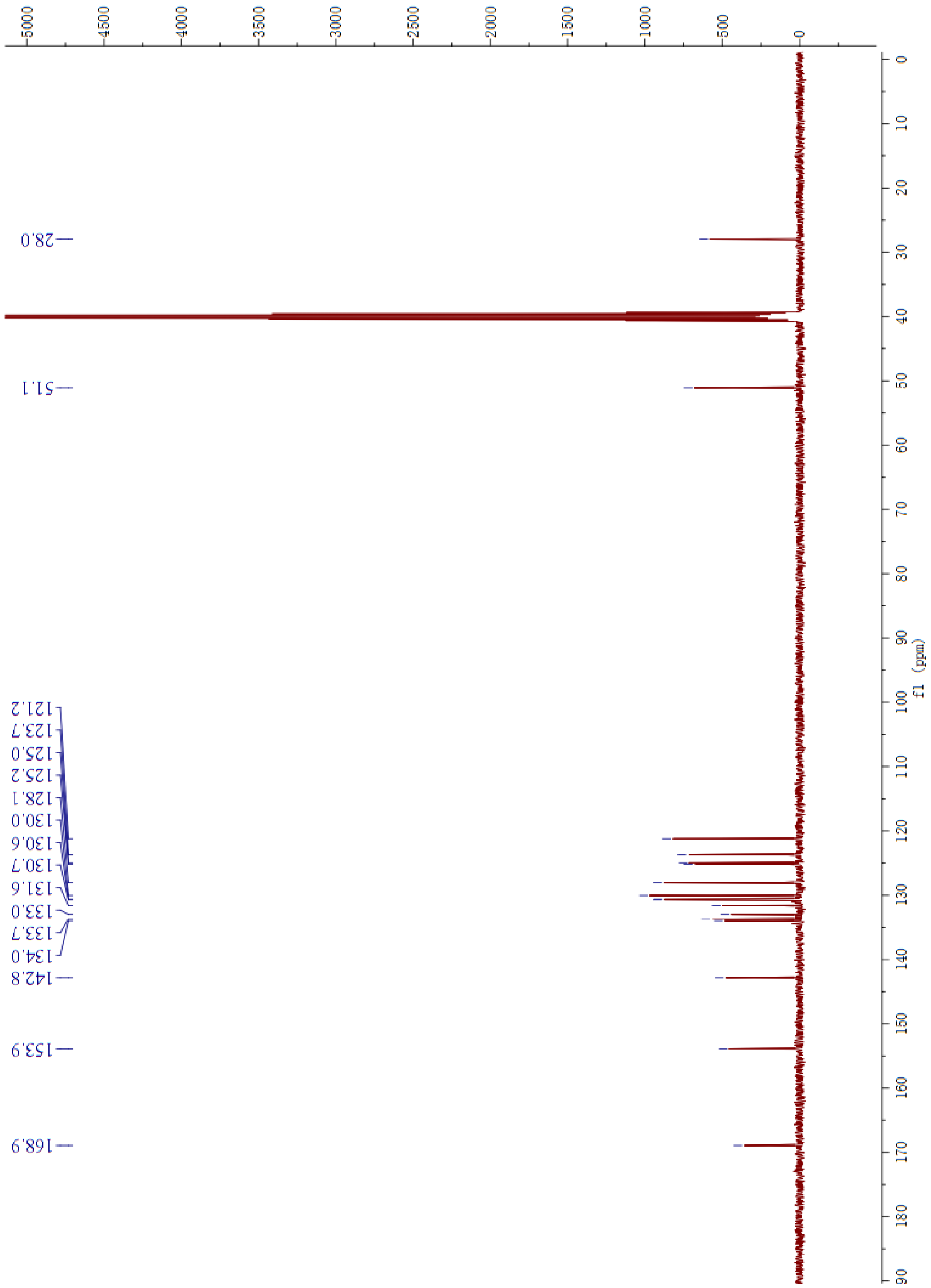

Figure S 24: ^{13}C NMR of compound 6l

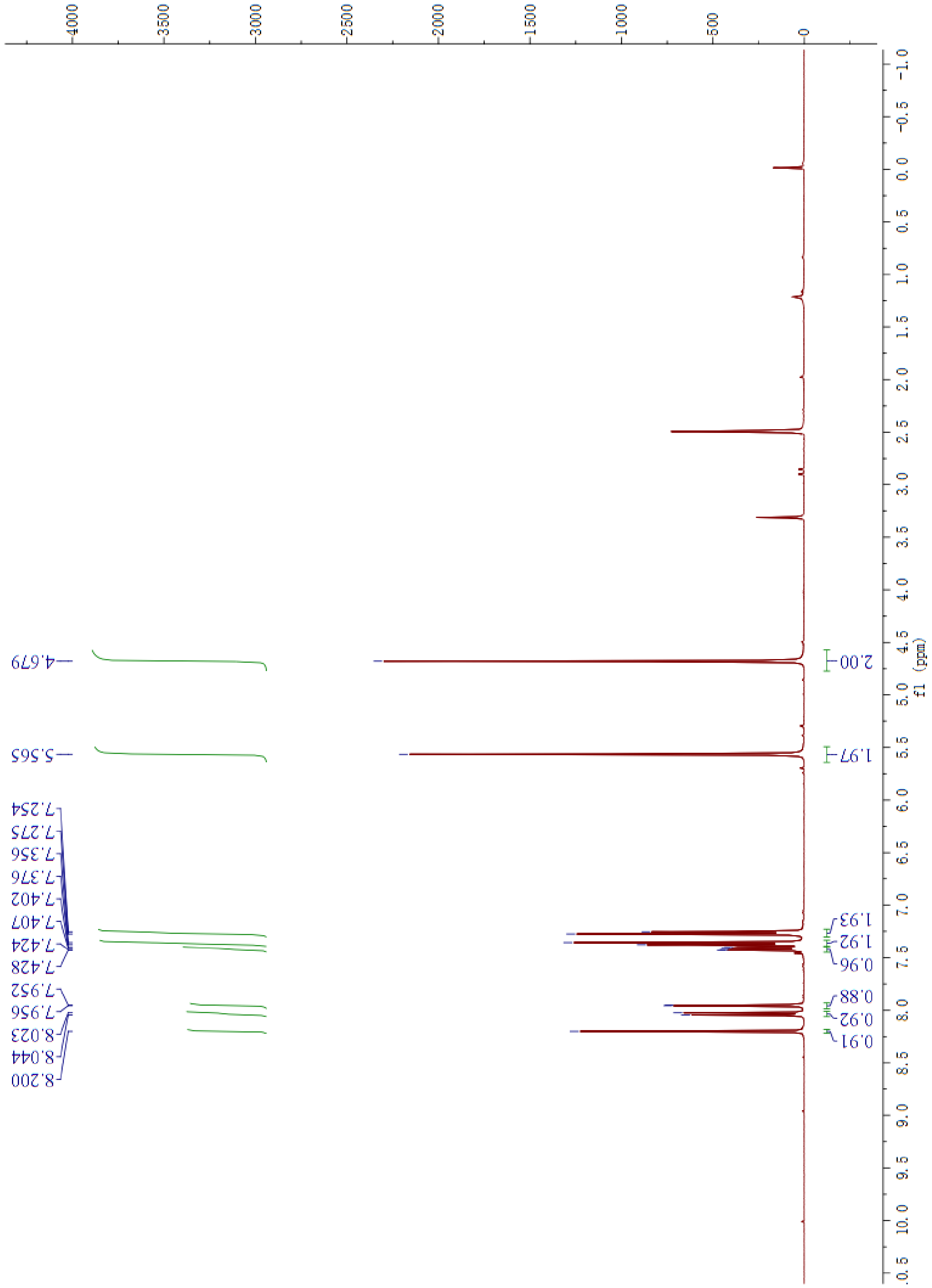

Figure S 25: ^{1}H NMR of compound 6m

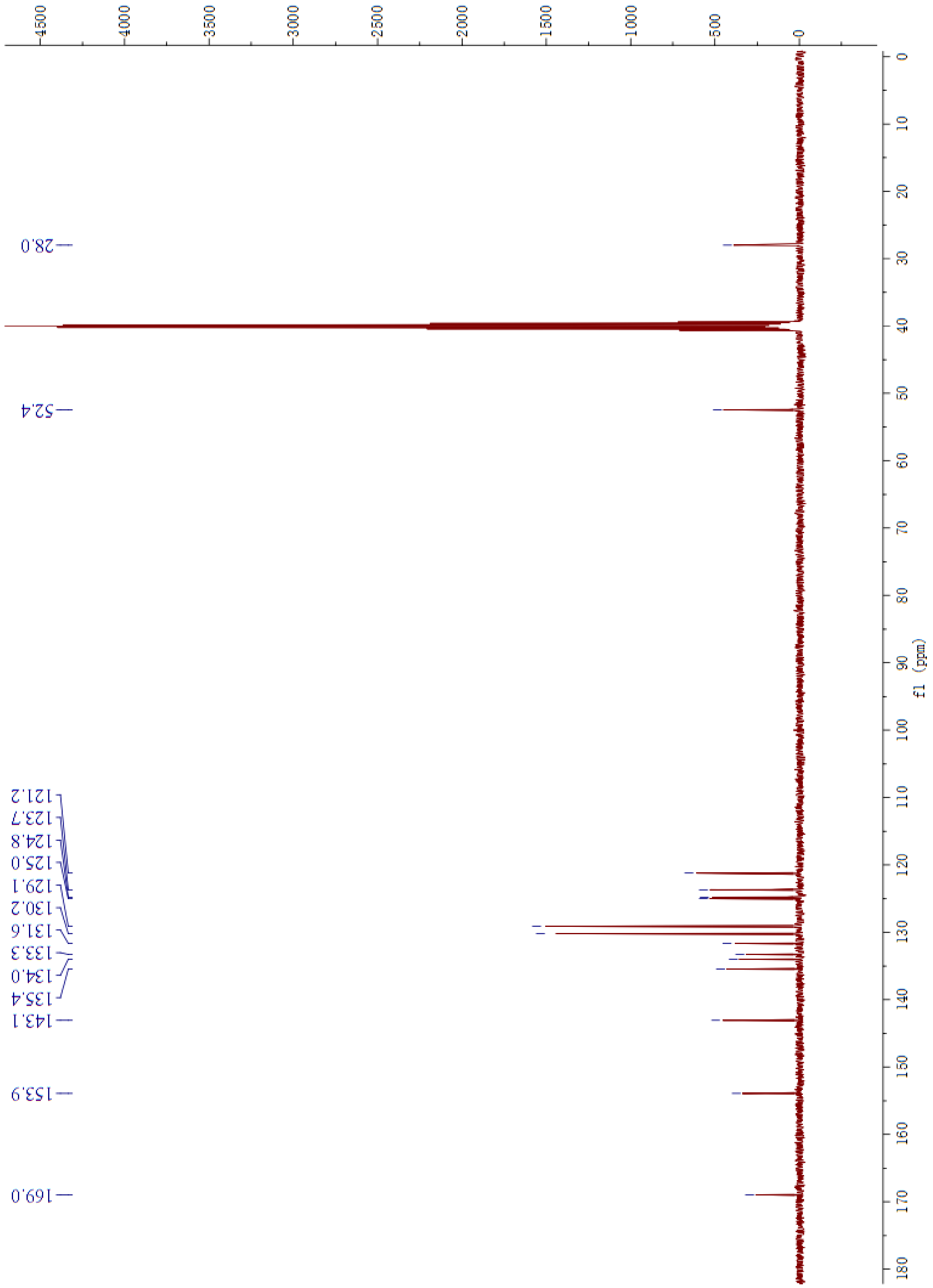

Figure S 26: ^{13}C NMR of compound 6m

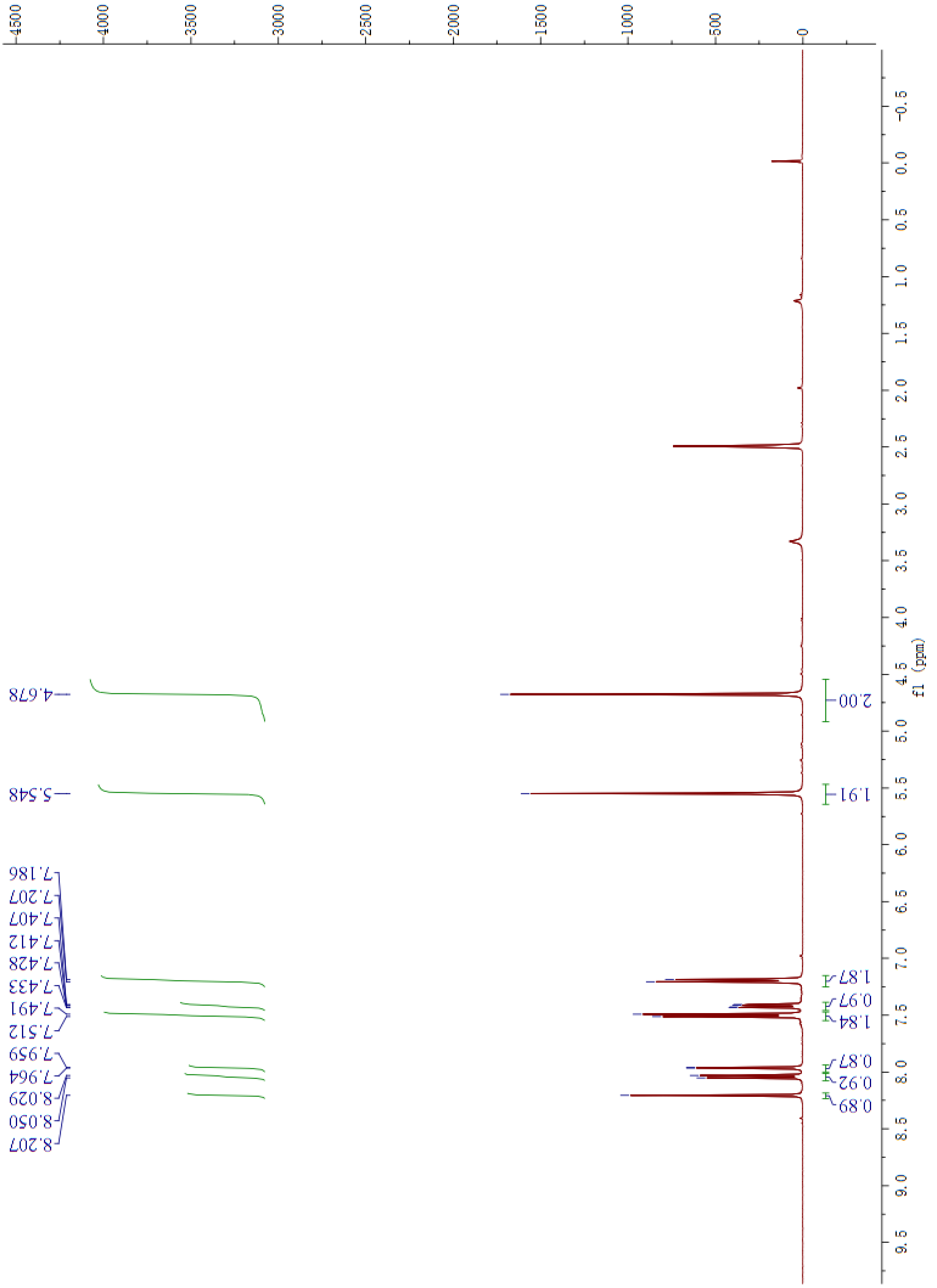

Figure S 27: ^{1}H NMR of compound 6n

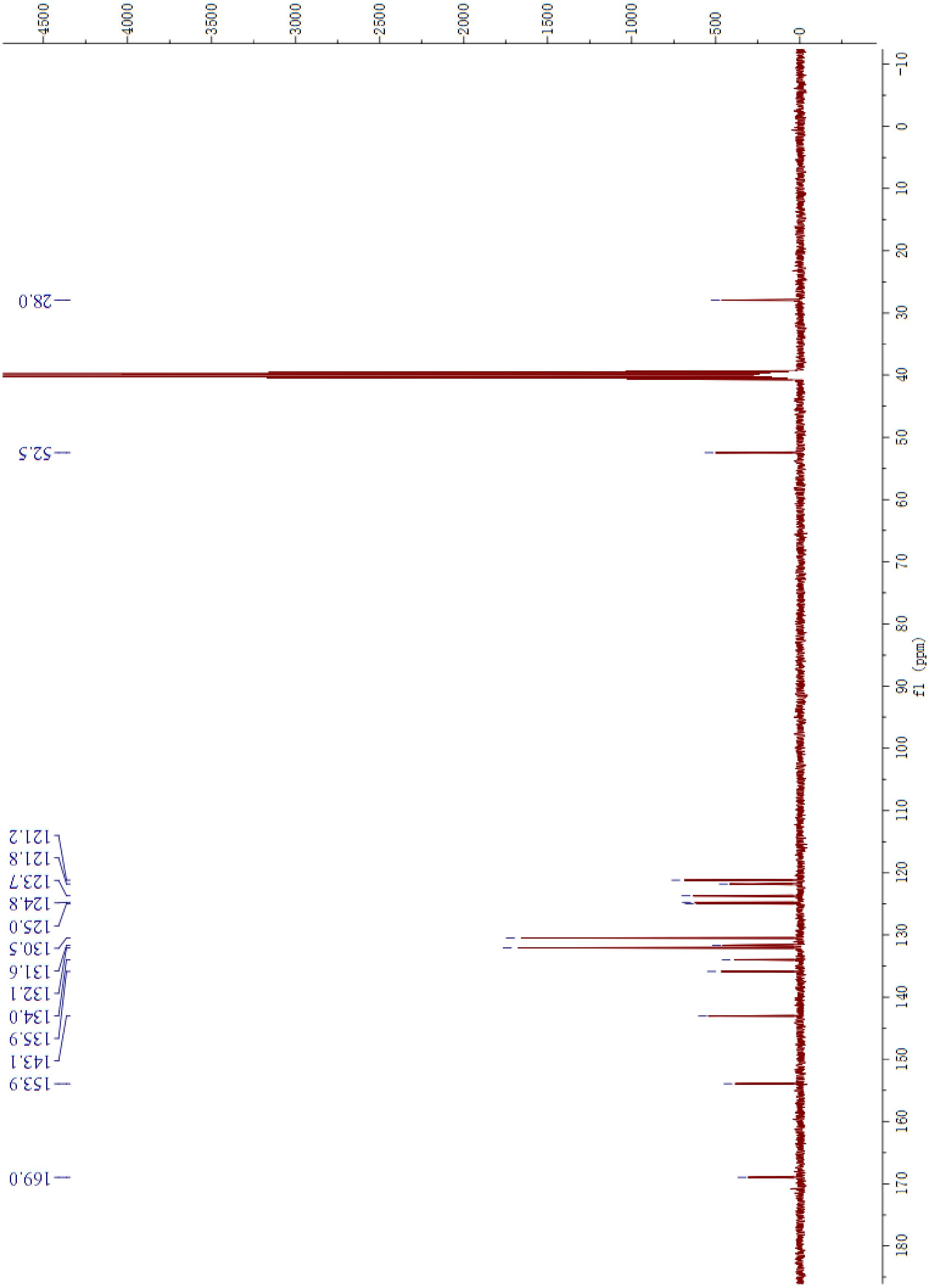

Figure S 28: ^{13}C NMR of compound 6n

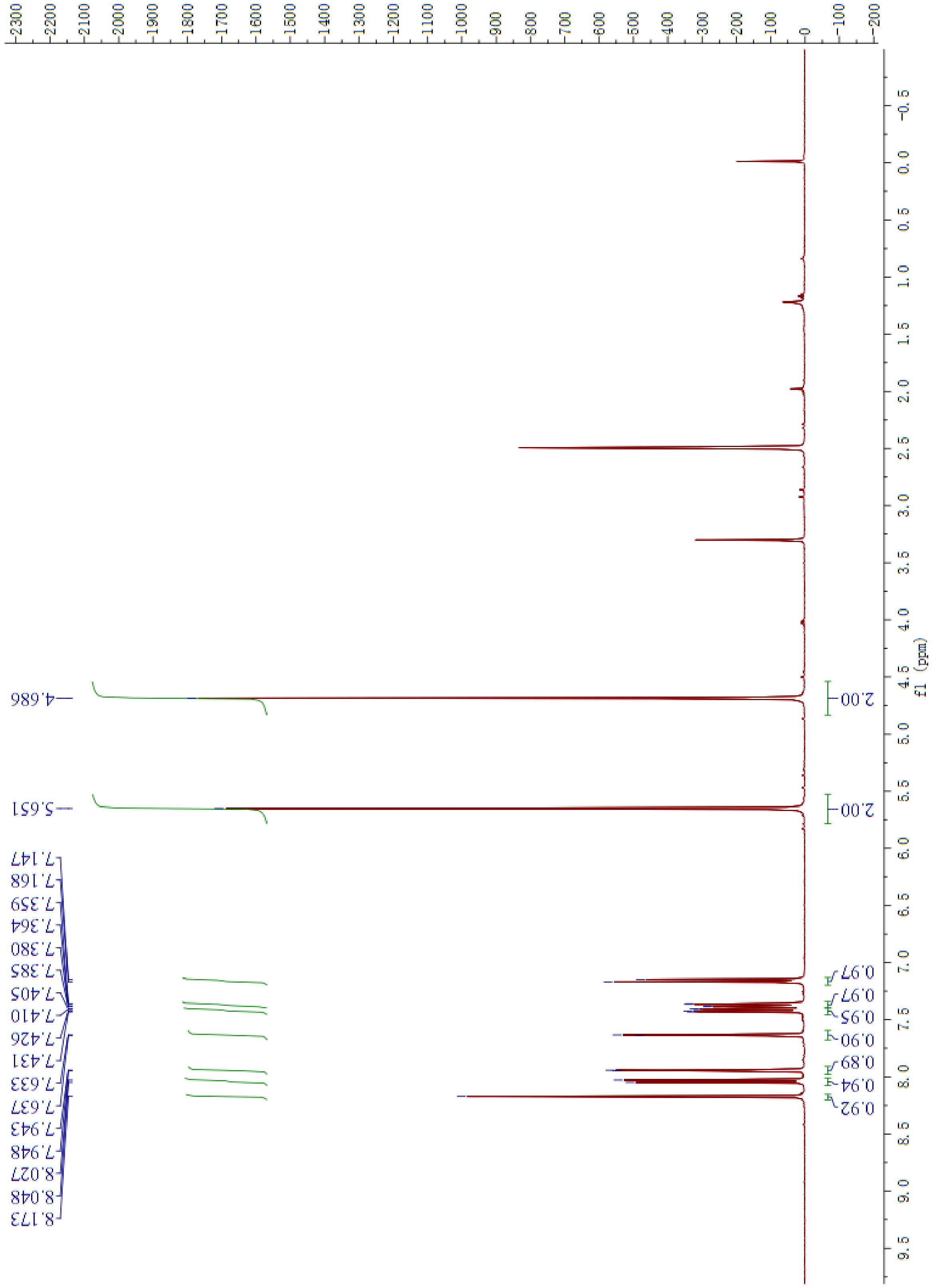

Figure S 29: ^{1}H NMR of compound 6o

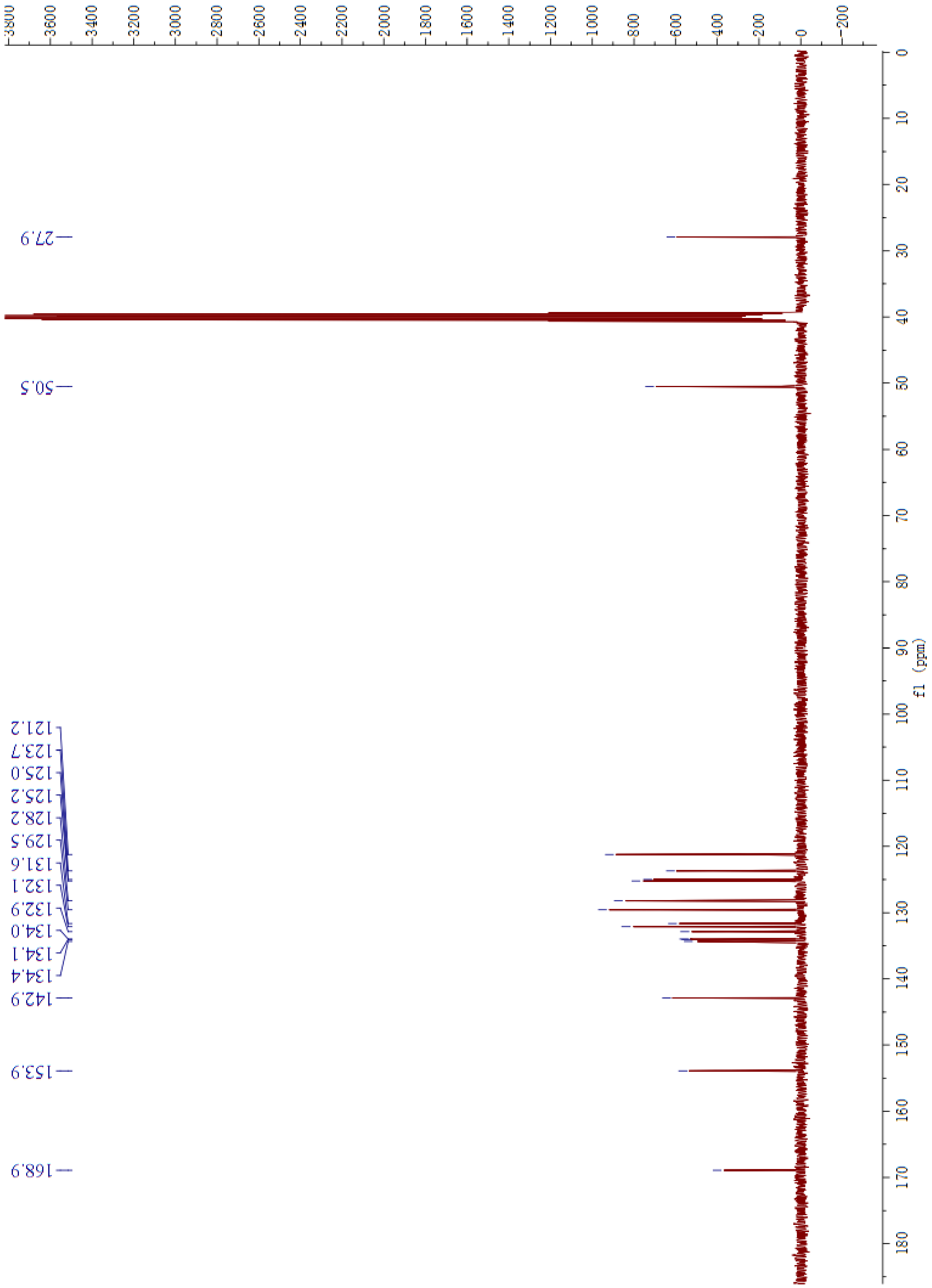

Figure S 30: ^{13}C NMR of compound 6o

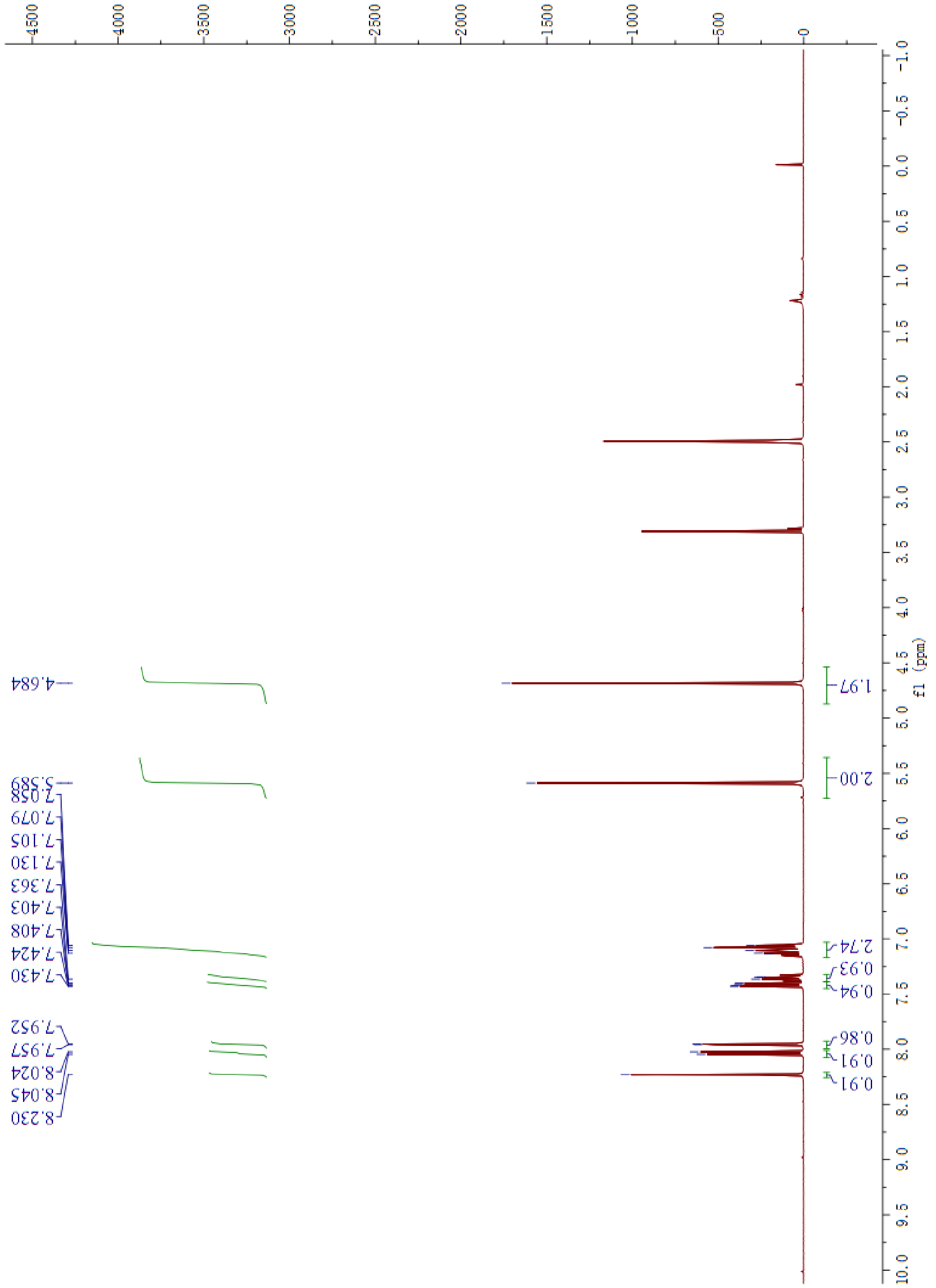

Figure S 31: ^{1}H NMR of compound 6p

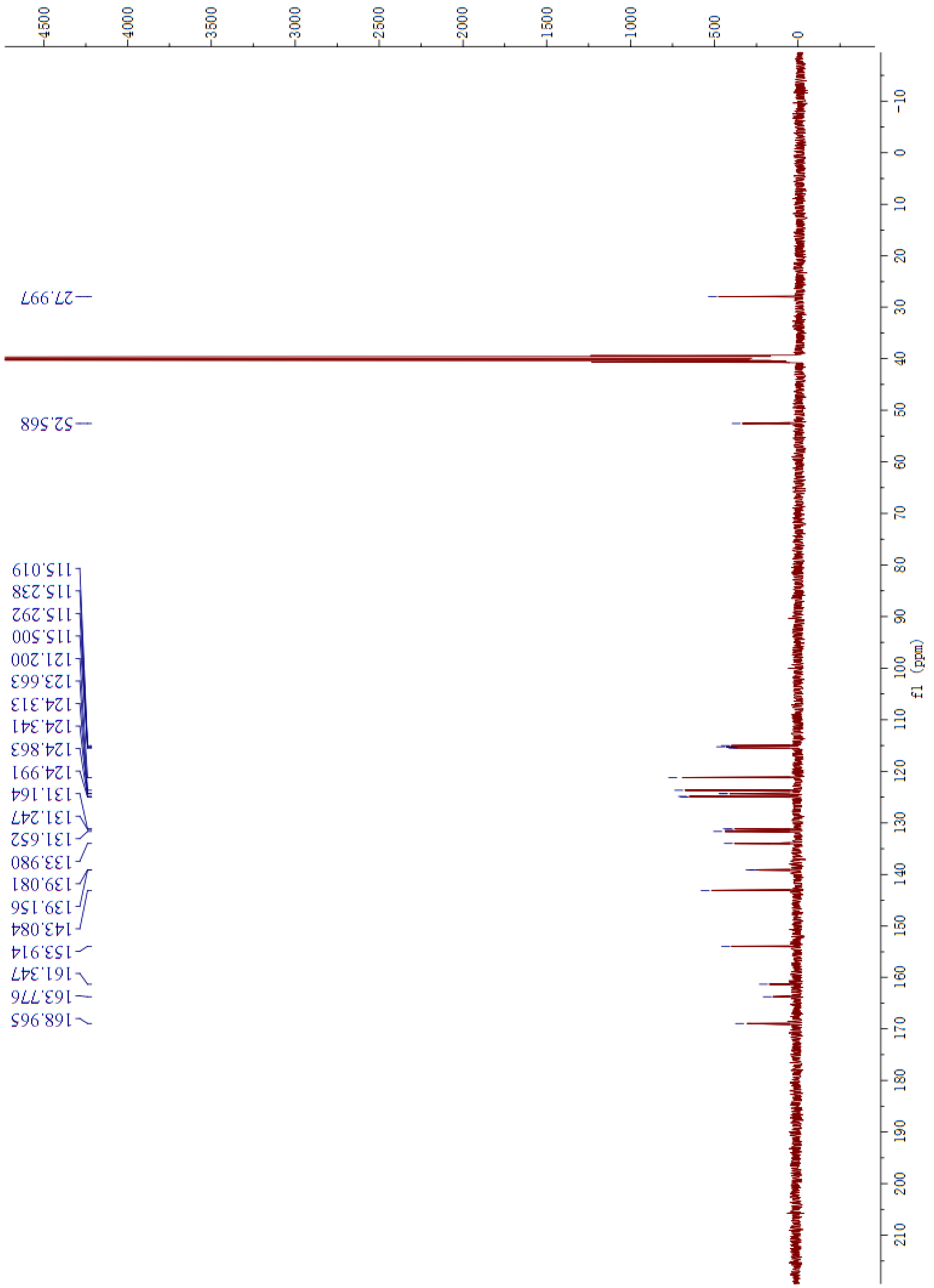

Figure S 32: ^{13}C NMR of compound 6p

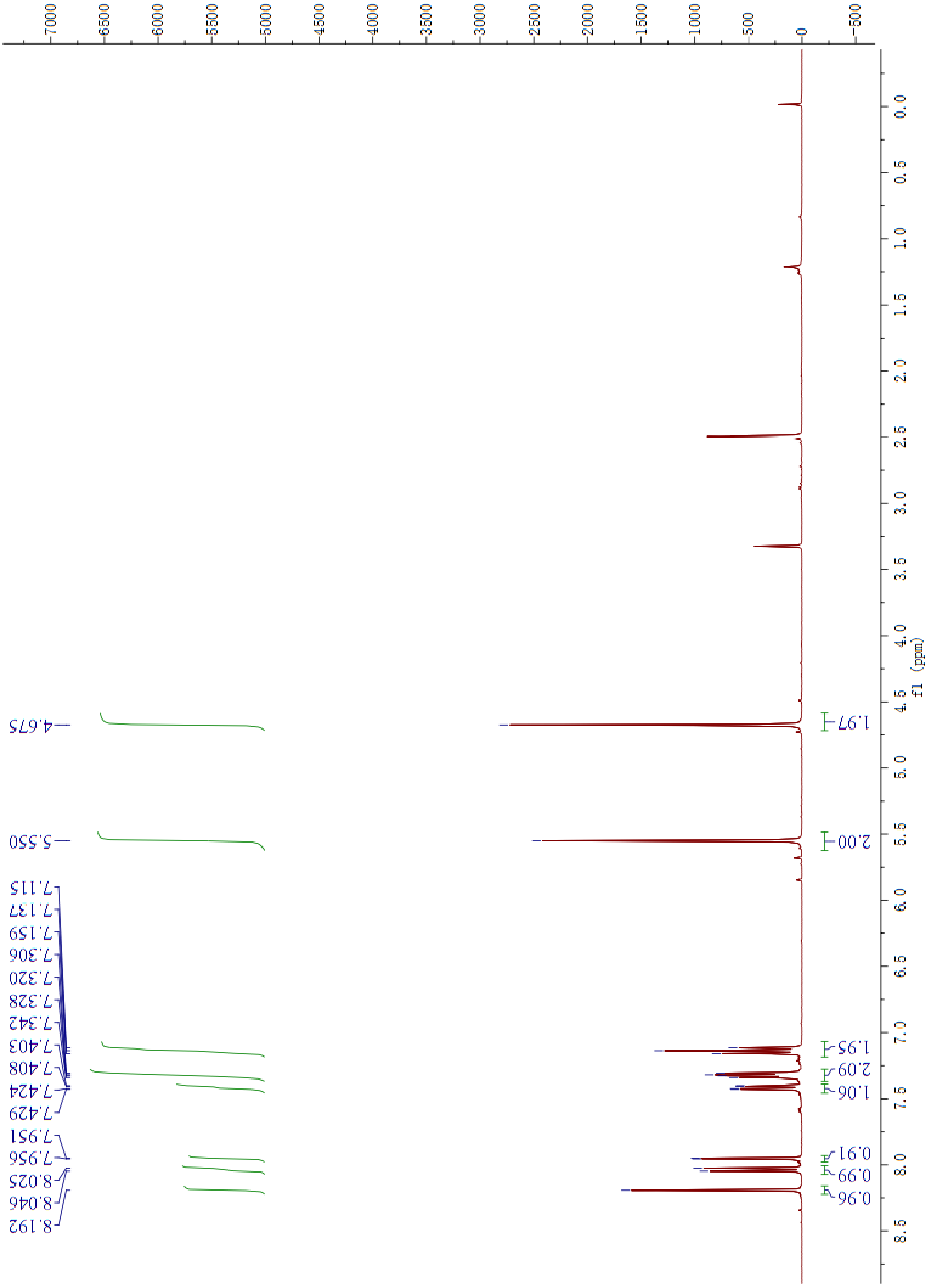

Figure S 33: ^{1}H NMR of compound 6q

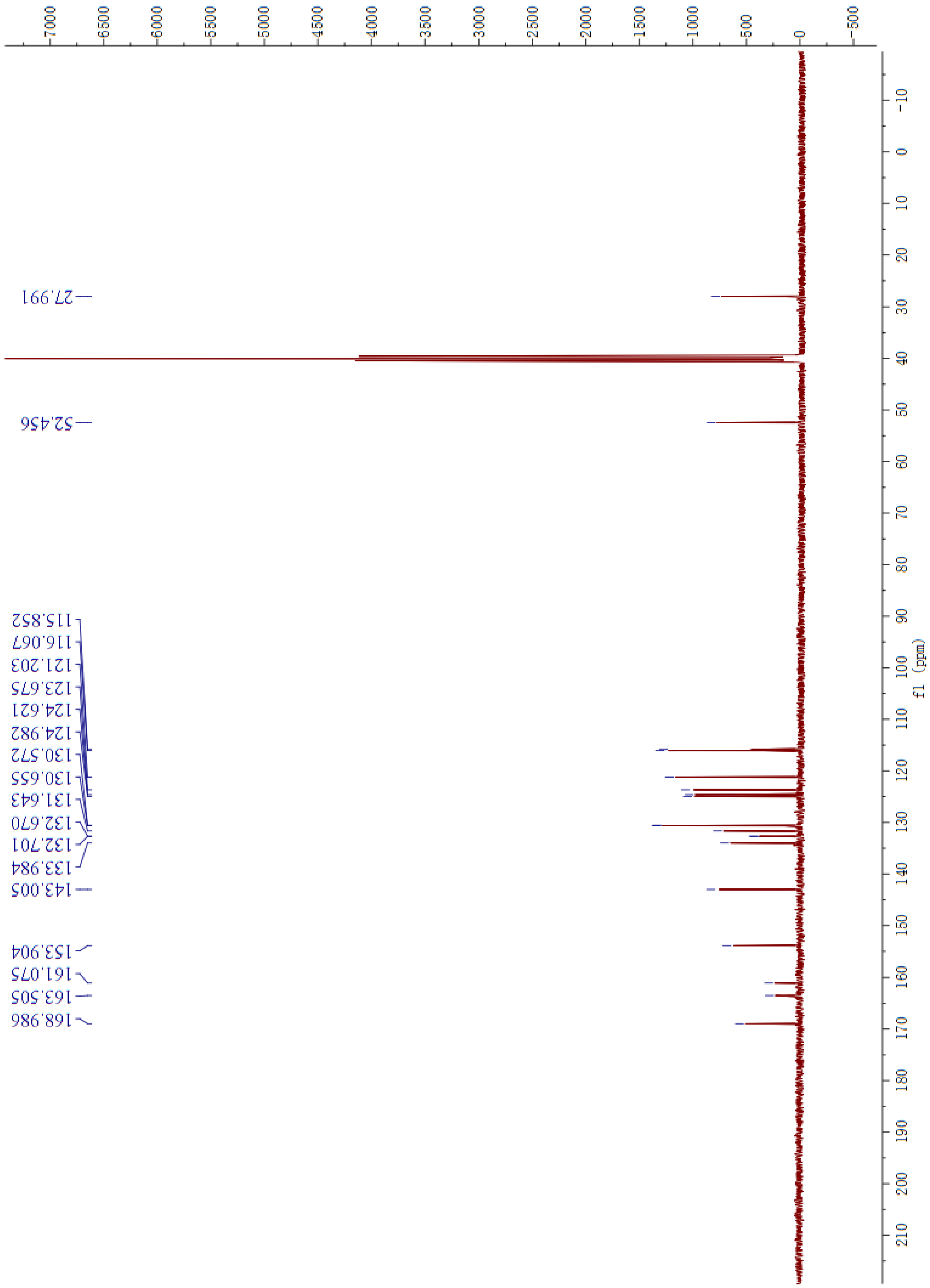

Figure S 34: ^{13}C NMR of compound 6q

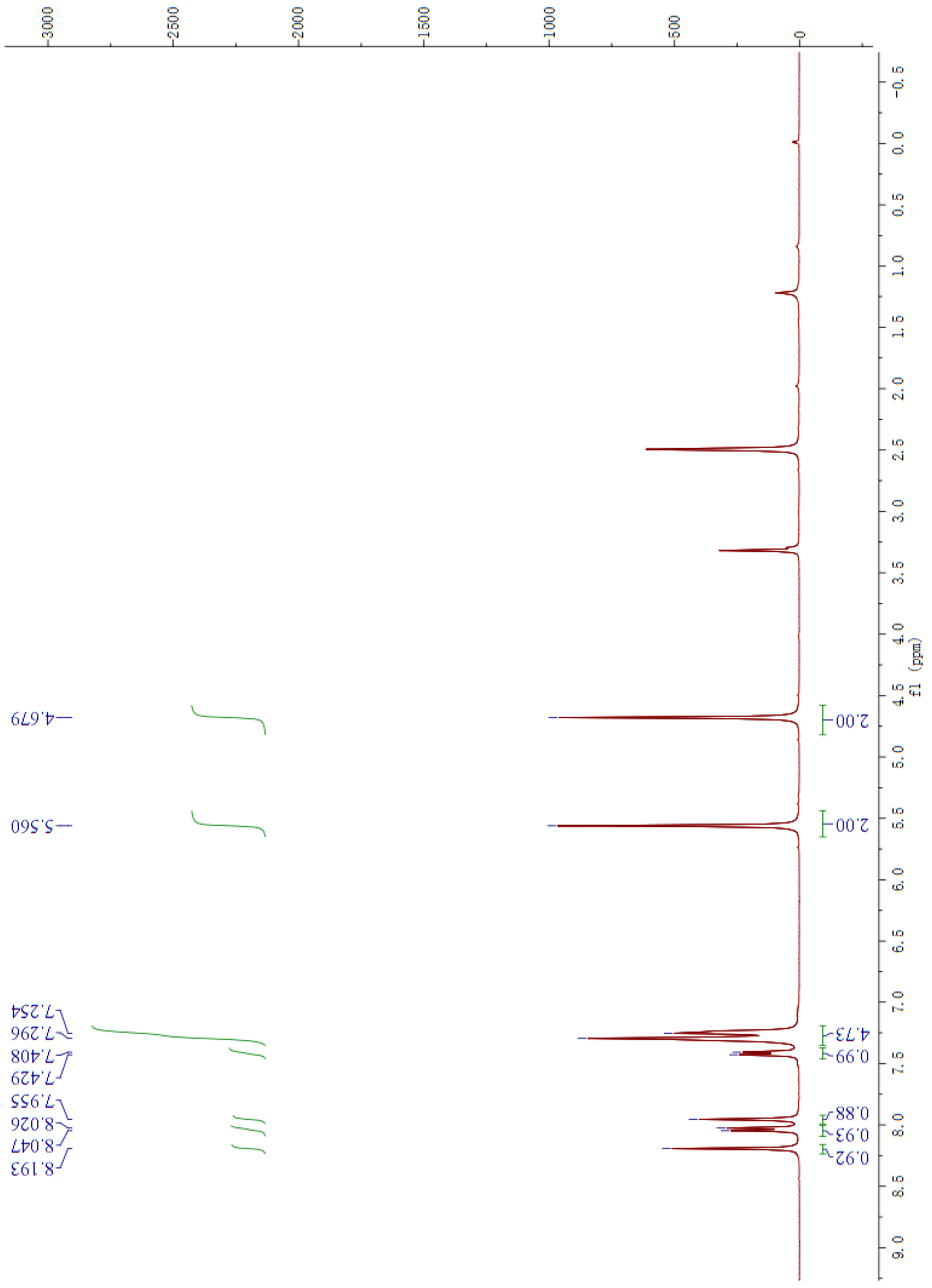

Figure S 35: ^{1}H NMR of compound 6r

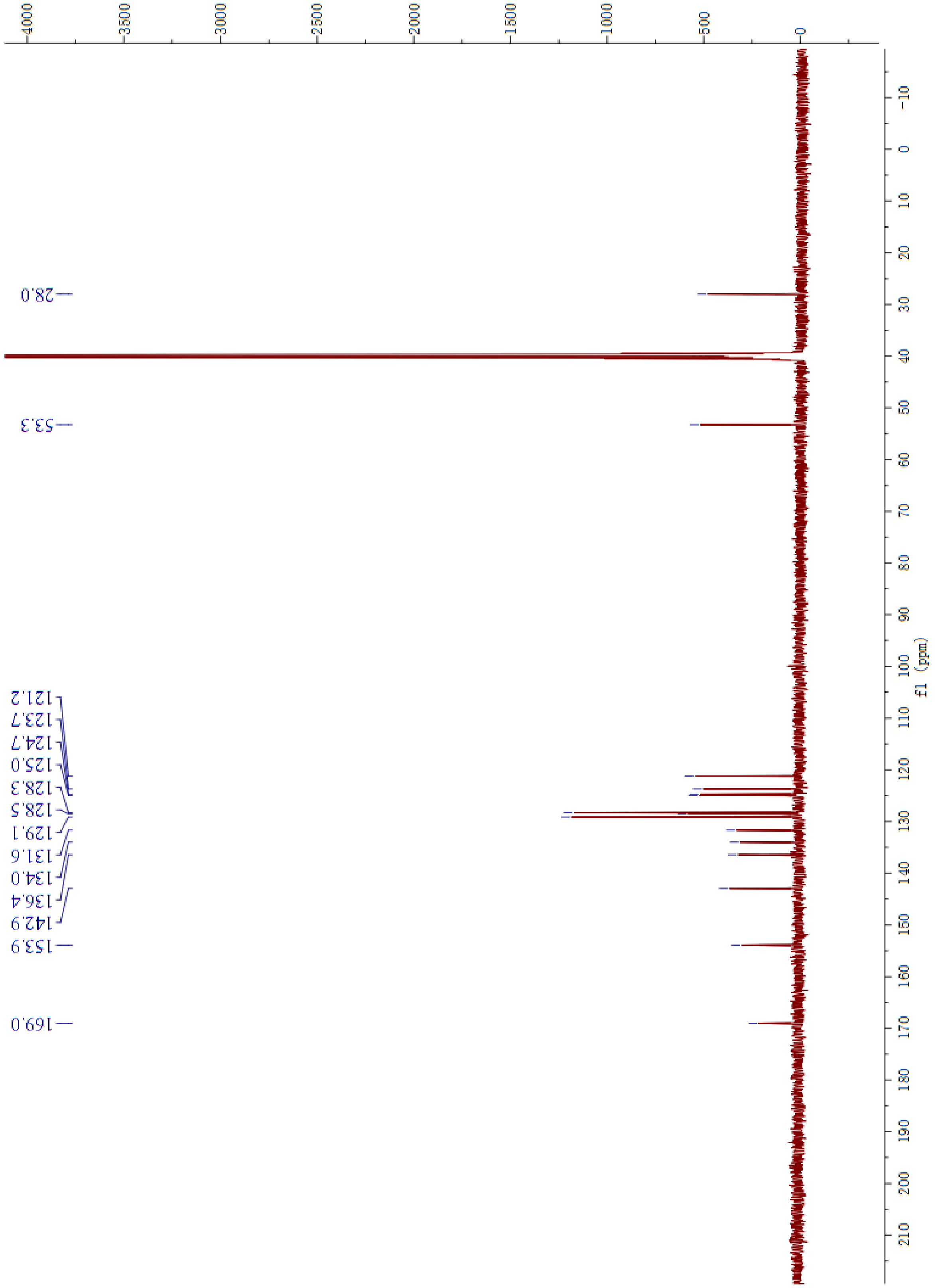

Figure S 36: ^{13}C NMR of compound 6r

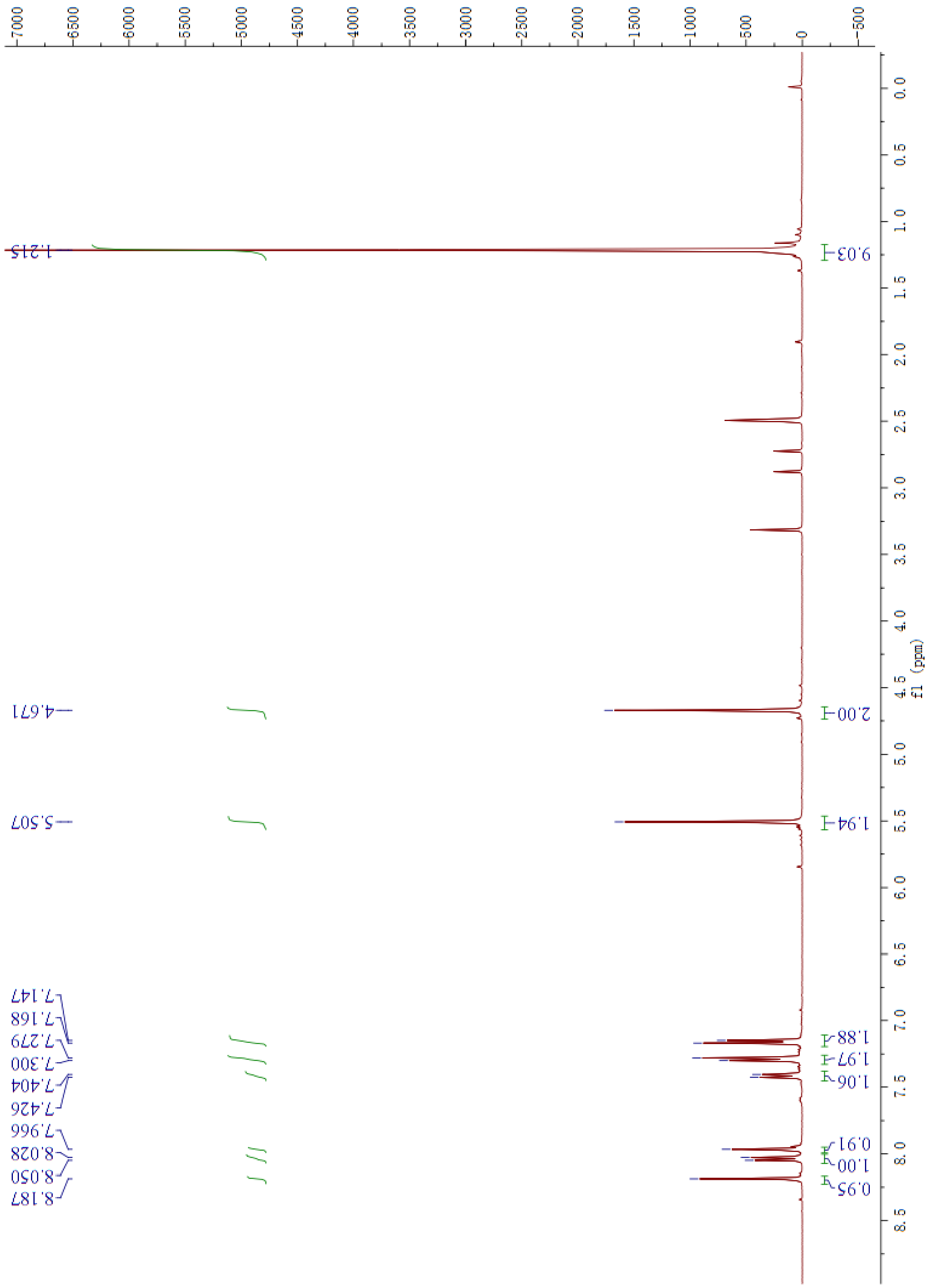

Figure S 37: ^{1}H NMR of compound 6s

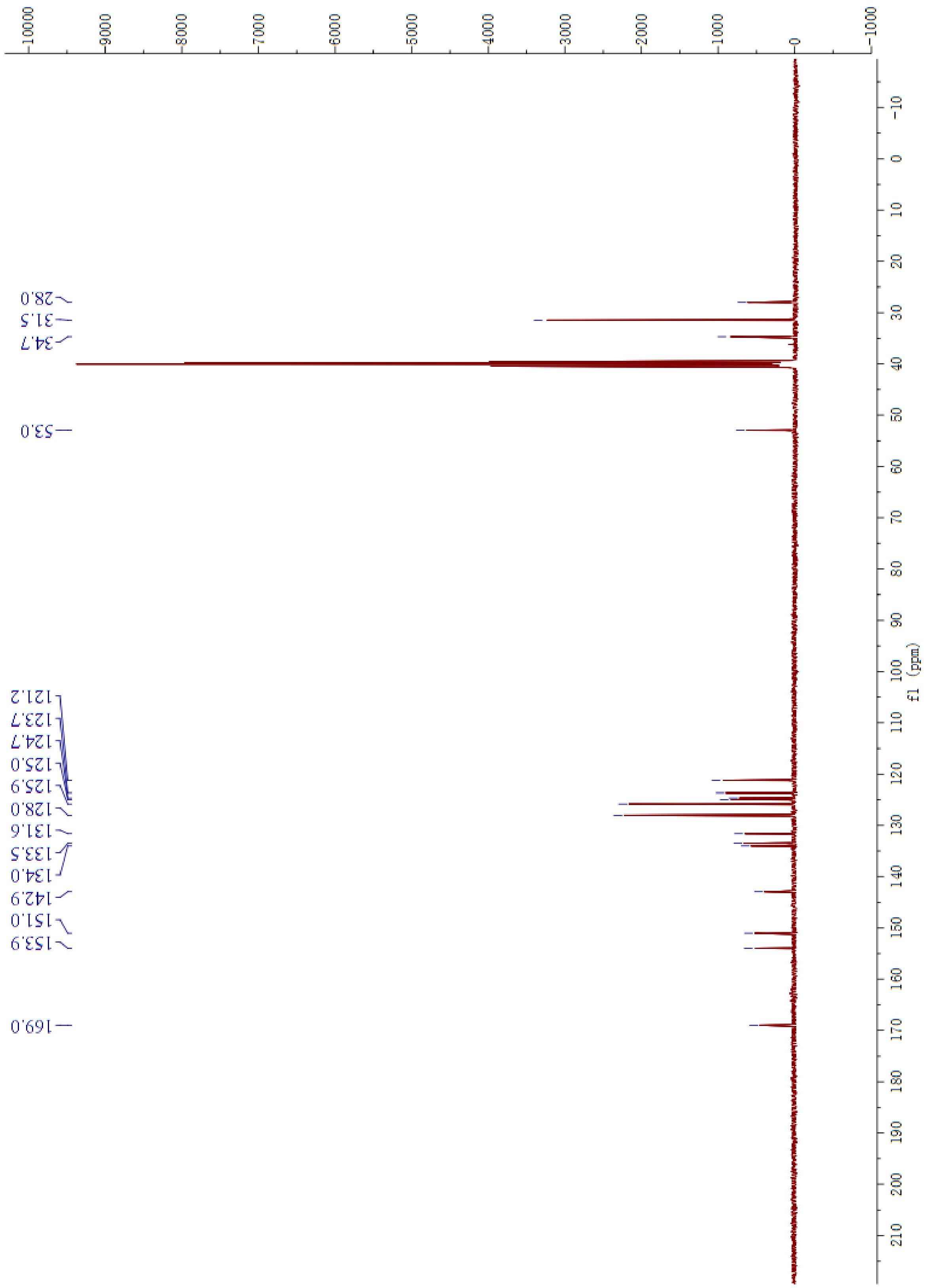

Figure S 38: ^{13}C NMR of compound 6s
